# Brain-wide mapping reveals that engrams for a single memory are distributed across multiple brain regions

**DOI:** 10.1038/s41467-022-29384-4

**Published:** 2022-04-04

**Authors:** Dheeraj S. Roy, Young-Gyun Park, Minyoung E. Kim, Ying Zhang, Sachie K. Ogawa, Nicholas DiNapoli, Xinyi Gu, Jae H. Cho, Heejin Choi, Lee Kamentsky, Jared Martin, Olivia Mosto, Tomomi Aida, Kwanghun Chung, Susumu Tonegawa

**Affiliations:** 1 RIKEN-MIT Laboratory for Neural Circuit Genetics at the Picower Institute for Learning and Memory, Department of Biology and Department of Brain and Cognitive Sciences, Massachusetts Institute of Technology, Cambridge, MA 02139 USA; 2 Broad Institute of MIT and Harvard, Cambridge, MA 02142 USA; 3 Institute for Medical Engineering and Science, Picower Institute for Learning and Memory, Department of Chemical Engineering and Department of Brain and Cognitive Sciences, Massachusetts Institute of Technology, Cambridge, MA 02139 USA; 4 Department of Bio and Brain Engineering, Korea Advanced Institute of Science and Technology (KAIST), Daejeon, South Korea; 5 McGovern Institute for Brain Research, Massachusetts Institute of Technology, Cambridge, MA 02139 USA; 6 Department of Psychiatry, University of Texas Southwestern Medical Center, Dallas, TX 75390 USA; 7 Yonsei-IBS Institute, Yonsei University, Seoul, 03722 Republic of Korea; 8 Howard Hughes Medical Institute, Massachusetts Institute of Technology, Cambridge, MA 02139 USA

**Keywords:** Fear conditioning, Long-term memory, Neural circuits, Cellular neuroscience

## Abstract

Neuronal ensembles that hold specific memory (memory engrams) have been identified in the hippocampus, amygdala, or cortex. However, it has been hypothesized that engrams of a specific memory are distributed among multiple brain regions that are functionally connected, referred to as a unified engram complex. Here, we report a partial map of the engram complex for contextual fear conditioning memory by characterizing encoding activated neuronal ensembles in 247 regions using tissue phenotyping in mice. The mapping was aided by an engram index, which identified 117 cFos^+^ brain regions holding engrams with high probability, and brain-wide reactivation of these neuronal ensembles by recall. Optogenetic manipulation experiments revealed engram ensembles, many of which were functionally connected to hippocampal or amygdala engrams. Simultaneous chemogenetic reactivation of multiple engram ensembles conferred a greater level of memory recall than reactivation of a single engram ensemble, reflecting the natural memory recall process. Overall, our study supports the unified engram complex hypothesis for memory storage.

## Abstract (web summary)

Where memories are located in our brains is not well understood. In this paper, the authors demonstrate that memories are spread out throughout multiple brain regions.

## Introduction

A memory engram is the enduring physical or chemical changes that occur in brain networks upon learning representing acquired memory information, and it is thought that recall is realized by the expression of these changes^1^. Neuronal ensembles that are activated by learning hold engrams and a reactivation of these neurons gives rise to recall of the specific memory^1–3^.

Following the initial demonstrations of memory engram cell ensembles^2,4,5^, studies have identified engram cell ensembles for several memories in different brain regions, such as hippocampal subfields^6,7^, amygdala subregions^8^, retrosplenial cortex^9^, and prefrontal cortex^10^. Semon’s engram concept as the storage site of memory suggested that a given memory is stored not in a single engram ensemble localized in a brain site but instead in a unified engram complex—a network of engram cell ensembles connected and distributed in dispersed brain regions^1,11,12^. Indeed, early limited studies supported this concept for contextual fear memory^4,5,9,10^. While these findings enhanced our understanding of engram-based memory storage, a thorough mapping of a unified engram complex for a specific memory has been a challenging endeavor.

The development of tissue clearing techniques, such as CLARITY^13^ and iDISCO^14^, combined with advanced microscopy has enabled high-throughput analyses of intact mouse brain samples. Brain-wide mapping of neurons activated upon learning in the form of the expression of an immediate early gene, for instance, cFos, so-called “brain-wide activity mapping” has been applied to haloperidol administration, whisker stimulation, and parental behavior^15^, foot shocks and cocaine administration^16^, and fear memory^17,18^. Some of these studies, however, did not ascertain whether the activated neurons satisfied the basic criteria for engram-holding ensembles^15,17^. In the other studies, engram ensembles were demonstrated only in a limited brain region namely in the prefrontal cortex rather than in a brain-wide manner^16,18^. To further our understanding of the organization of memory engram cell ensembles, the experiment should be designed in the first step to identify neuronal activity patterns induced by memory formation in a more holistic manner. Since the recall-induced reactivation of neuronal ensembles that was previously activated by learning is also a crucial criterion of an engram cell ensemble^1–3^, it is essential for engram mapping studies to demonstrate this property.

In the present study, we identified engram cell ensembles and their high probability candidates for a contextual fear memory, by following a four-step approach. First, we applied in a significantly broader set of brain regions SHIELD-based tissue phenotyping^19^ for brain-wide mapping of activated neurons using the Fos-TRAP mouse line^20^. Using an unbiased and automated approach, we cross-compared the activity of 247 brain regions during contextual fear memory encoding and recent memory recall. Second, we devised an engram index to rank-order putative engram cell ensemble candidates because conducting optogenetic or chemogenetic manipulations that permit an assertion of engrams in hundreds of brain regions that display activity-dependent labeling is impractical. Third, we performed brain-wide engram reactivation studies, which revealed engrams that are reactivated by recall. Fourth, focusing on a dozen significant brain regions, narrowed down by the engram index and the engram reactivation experiments, we conducted optogenetic manipulations. This not only confirmed previously identified engrams but also revealed engrams in additional brain regions. Furthermore, many of these engrams and engram candidates were functionally connected to hippocampal CA1 or basolateral amygdala engrams. Finally, simultaneous reactivation of multiple engram cell ensembles using chemogenetics conferred greater levels of memory recall than reactivation of their subsets, as would be expected from the natural process of memory recall. Together, our study supports Semon’s unified engram complex hypothesis^1^ that a specific memory is stored in functionally connected engram cell ensembles that are widely distributed in multiple brain regions.

## Results

### Brain-wide 3D imaging and mapping of activated neurons

**Fig. 1:**
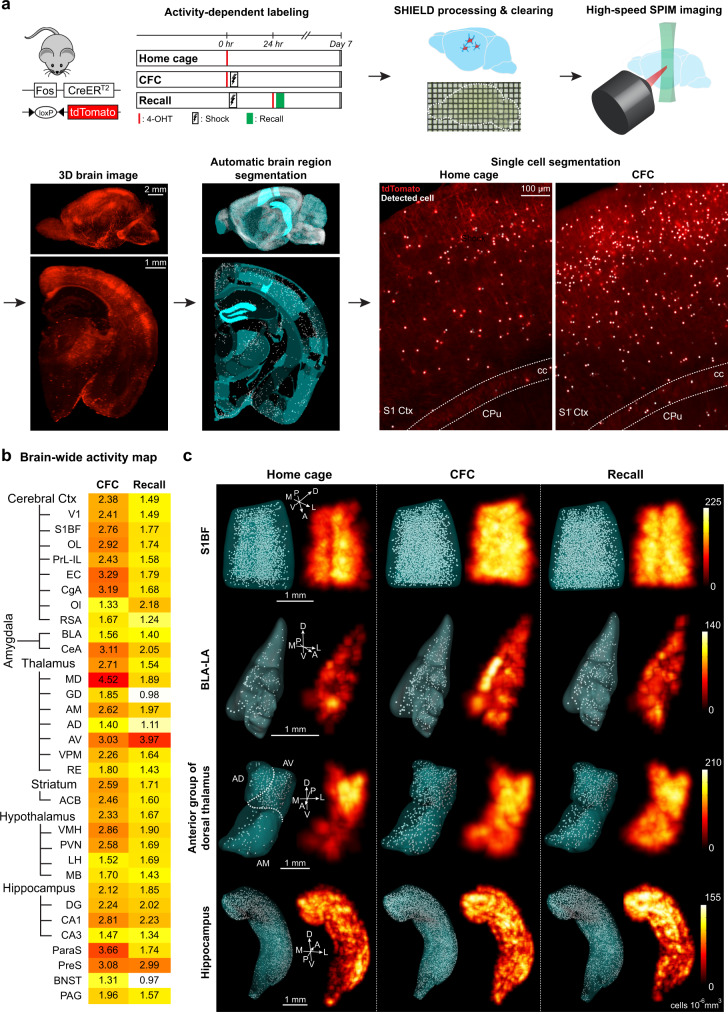
Generation of behavior epoch-specific brain-wide activity maps. **a** Activity mapping pipeline. We used Fos-TRAP mice crossed with a Cre-dependent tdTomato reporter mouse line to prepare three behavioral cohorts: home cage, contextual fear conditioning (CFC), and fear memory recall (Recall). Brain samples were used for SHIELD processing, SPIM imaging, 3D reconstructions, automatic brain region segmentation, and automatic single activated-cell detection. Primary somatosensory cortex (S1 Ctx), corpus callosum (cc), caudate putamen (CPu). **b** Brief, representative version of our brain-wide activity mapping results (*n* = 7 home cage mice, *n* = 10 CFC mice, *n* = 9 Recall mice), where the behavioral groups are normalized to home cage data. Individual values indicate fold increases in the numbers of activated neurons relative to home cage. Primary visual area (V1), primary somatosensory area barrel field (S1BF), orbital cortex (OL), prelimbic cortex (PrL), infralimbic cortex (IL), entorhinal cortex (EC), anterior cingulate cortex (CgA), olfactory cortex (Ol), retrosplenial cortex (RSA), basolateral amygdala (BLA), central amygdala (CeA), mediodorsal thalamus (MD), dorsal geniculate thalamus (GD), anteromedial thalamus (AM), anterodorsal thalamus (AD), anteroventral thalamus (AV), ventroposterior medial nucleus of thalamus (VPM), nucleus reuniens of thalamus (RE), nucleus accumbens (ACB), ventromedial hypothalamus (VMH), paraventricular hypothalamus (PVN), lateral hypothalamus (LH), mammillary body (MB), dentate gyrus (DG), hippocampal CA1 (CA1), hippocampal CA3 (CA3), para-subiculum (ParaS), pre-subiculum (PreS), bed nucleus of the stria terminalis (BNST), and periaqueductal gray (PAG). For a full list of brain regions analyzed, refer to Supplementary Tables 1 and 2. **c** 3D rendering of four brain regions, specifically S1BF, BLA-LA, anterior group of dorsal thalamus, and hippocampus, showing automatically segmented activated neuronal populations along with corresponding heat maps. LA lateral amygdala, D dorsal, V ventral, A anterior, P posterior, M medial, L lateral.

The first step of our unbiased engram mapping approach required brain-wide 3D imaging and mapping of activated neurons using the Fos-TRAP mouse line and SHIELD. Expression of immediate-early genes (IEGs) has been used to visualize neuronal activity in a given brain region^21^. By linking the expression of Cre^ERT2^ to the IEG cFos in a tamoxifen-dependent manner, the Fos-TRAP mouse line permits brain-wide labeling of activated neurons within a user-defined time window of several hours^20^. We crossed Fos-TRAP mice with a Cre-dependent tdTomato reporter mouse line (Fig. 1a). Using the fast-acting 4-hydroxytamoxifen (4-OHT), we prepared three behavioral cohorts: mice that received 4-OHT and remained in their home cage for the labeling duration (home cage group), mice that received 4-OHT followed by contextual fear conditioning training and were returned to their home cage (CFC group), and mice that received 4-OHT followed by fear memory recall 24 h after CFC and were returned to their home cage (recall group). One week after labeling, brains were SHIELD-processed^19^ to preserve endogenous tdTomato fluorescence and tissue architecture for the clearing process. The optically transparent SHIELD brains were imaged using a custom-built high-speed selective plane illumination microscope (SPIM) (Supplementary Fig. 1 and Supplementary Movie 1 and 2). Due to the high quality of tissue architecture preservation during the clearing and imaging processes, 3D brain images could be automatically aligned to a standard mouse brain atlas, and further, we could successfully perform automatic brain region segmentation. Crucially, by applying a neural network-based automatic cell counting algorithm, we detected tdTomato-positive activated cells in the entire brain sample at single cell resolution; representative examples from home cage and CFC groups are shown in Fig. 1a. These experiments validated our brain-wide activity-dependent mapping strategy.

### Generation of behavior epoch-specific brain-wide activity (cFos) maps

We quantified the number of activated neurons in 247 brain regions (Supplementary Tables 1 and 2) from home cage, CFC, and recall groups. To generate brain-wide activity maps, the tdTomato (i.e., cFos^+^) cell counts of CFC and recall groups were normalized by home cage cell counts (Fig. 1b). In the example activity map (Fig. 1b), we plotted the activation pattern for brain structures including cerebral cortex, amygdala, thalamus, striatum, hypothalamus, hippocampus, and their respective sub-divisions. While memory recall activated fewer neurons as compared to memory encoding, brain regions that were most strongly activated by recall were those that were also strongly activated by encoding (Supplementary Fig. 2a, b). This observation is consistent with the notion that memory engram ensembles that are activated by learning are reactivated during recall of the specific memory^1,2^.

Among the most robustly activated brain regions by memory encoding were the entorhinal cortex (EC), anterior cingulate cortex (CgA), amygdala, mediodorsal thalamus (MD), hippocampus, and para-subiculum (ParaS) (Fig. 1b, c). Importantly, as it has been reported that these structures play a crucial role in contextual fear learning and memory^22^, this activation pattern supports the accuracy of our brain-wide activity-mapping results. Other than these well-known learning-activated brain regions, two hypothalamic nuclei, ventromedial hypothalamus (VMH) and paraventricular hypothalamus (PVN), were activated by CFC encoding. Further, several areas including olfactory cortex (Ol), lateral hypothalamus (LH), and anteroventral thalamus (AV) showed higher activation in memory recall as compared to memory encoding.

### Identification of putative engram-containing brain regions

In the brain-wide activity maps, we found several regions where the neurons were strongly activated by both encoding and recall, but their role in memory formation has previously been less understood as compared to hippocampal and amygdala subregions (Fig. 1b). These regions include thalamic structures, namely the anteromedial thalamus (AM) sub-division of the anterior group of dorsal thalamus, MD, and nucleus reuniens thalamus (RE), as well as the striatum, para-subiculum (ParaS), and pre-subiculum (PreS).

**Fig. 2:**
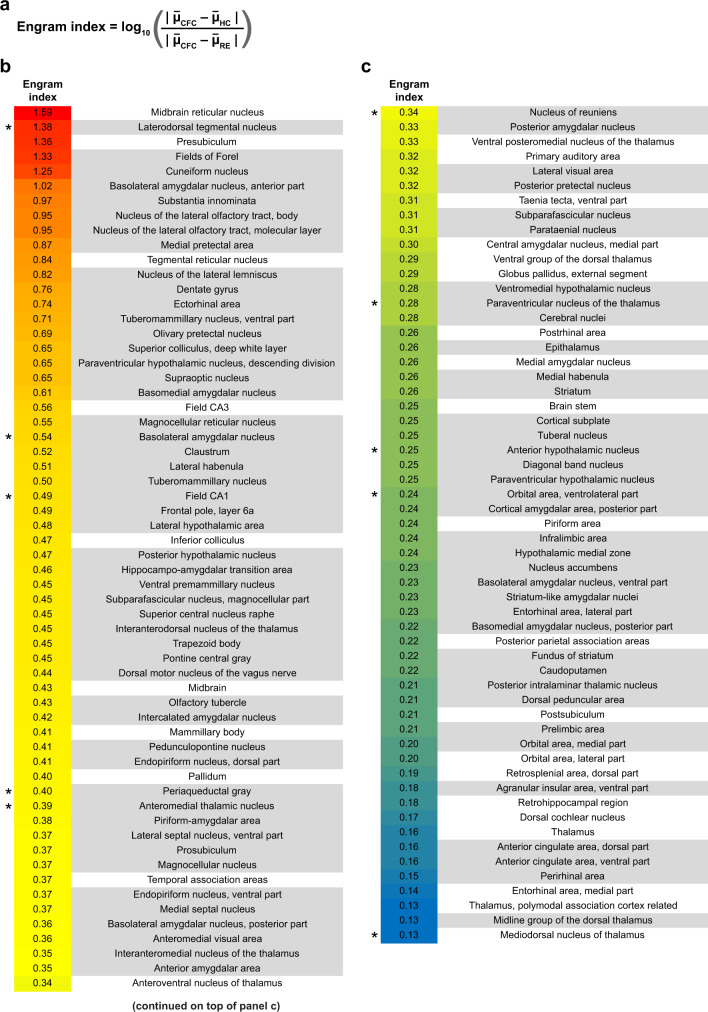
Brain-wide engram indices. **a** Engram index equation. Home cage (HC), CFC, and Recall (RE) groups. **b**, **c** Rank-ordered list of 117 brain regions with engram indices that passed our statistical criteria (Supplementary Table 1) (*n* = 7 home cage mice, *n* = 10 CFC mice, *n* = 9 Recall mice). For a list of 130 non-significant brain regions based on our statistical criteria, refer to Supplementary Table 2. Regions highlighted in gray have also been identified as exhibiting significant engram reactivation in Fig. 3. Regions with an asterisk were tested in the optogenetic engram reactivation behavioral experiments described in Fig. 4.

In order to obtain a list of putative engram-containing brain regions, we first narrowed down the list of analyzed brain regions by identifying those in which the cFos^+^ cell counts of CFC and recall were individually significantly higher than home cage (hereafter referred to as “significant brain regions”). We next created an engram index (Fig. 2a), calculated the index values only for significant brain regions, and rank-ordered these regions (Fig. 2b, c and Supplementary Tables 1 and 2). This index provides an unbiased criterion to identify brain regions that potentially carry memory engram cells. Hippocampal subdivisions, amygdala, and association cortical brain structures were among the significant regions with relatively high engram index values (Fig. 2b, c), supporting the validity of this engram index approach. In addition to these brain regions that have previously been demonstrated to have learning and memory functions, significant brain regions included midbrain structures (e.g., superior colliculus) and brainstem nuclei (e.g., reticular formation).

### Overlap of cFos^+^ cells between learning and recall

**Fig. 3:**
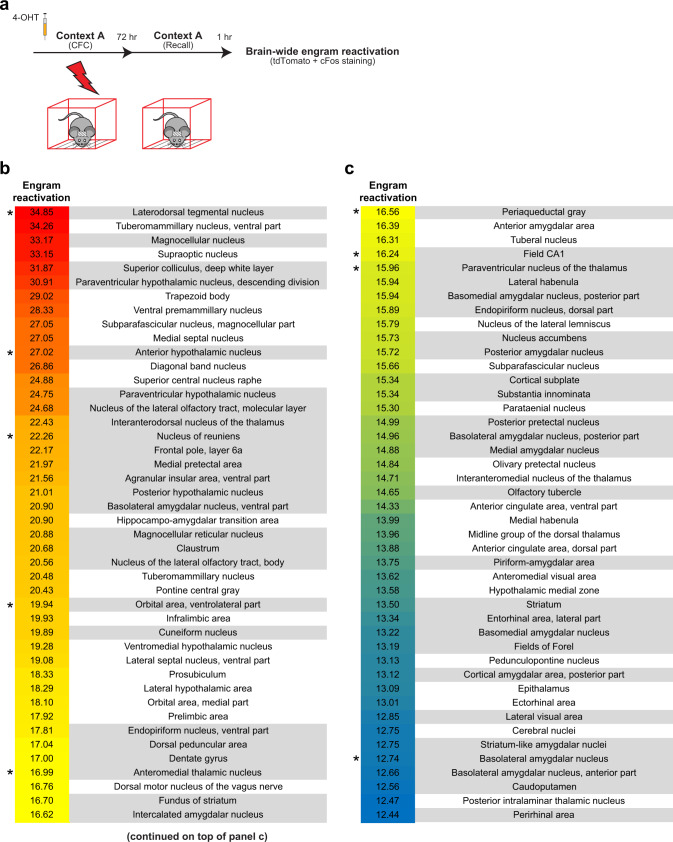
Brain-wide engram reactivation. **a** To identify CFC activated neurons that are reactivated by recall (i.e., exhibiting overlap), activated neurons during CFC were labeled using 4-OHT on day 1 and three days later activated neurons during recall were labeled using cFos staining. **b**, **c** Rank-ordered list of 88 brain regions with their engram reactivation values (percentage of CFC activated neurons that subsequently showed recall-induced cFos) that passed our statistical criteria (*n* = 9 CFC mice). For a list of 159 non-significant brain regions based on our statistical criteria, refer to Supplementary Table 3. Regions highlighted in gray are consistent with those identified by the brain-wide engram index analysis in Fig. 2. Regions with an asterisk were tested in the optogenetic engram reactivation behavioral experiments described in Fig. 4.

Because it is thought that learning-activated ensembles are reactivated during successful memory retrieval^1,2^, in order to build on the engram index measure, we performed brain-wide engram reactivation experiments. For this purpose, on day 1, CFC-activated ensembles were labeled with tdTomato, and three days later these mice received a recall test session (Fig. 3a). After SHIELD-processing, intact brain hemispheres were stained with a cFos antibody using the eFLASH technology^23^ and imaged using the SPIM. By examining the overlap of tdTomato and recall-activated cFos^+^ neurons, we generated a rank-ordered list of brain regions that exhibited engram reactivation above chance levels (Fig. 3b, c and Supplementary Fig. 2c and Supplementary Table 3). These experiments not only revealed significant engram reactivation in known hippocampal and amygdala regions, but also showed reactivation in many thalamic, cortical, midbrain, and brainstem structures (Fig. 3b, c). Importantly, when we compared the brain regions identified by the engram index analysis with these reactivated regions, we observed that ~60% of the regions were consistent between analyses (regions highlighted in gray in Figs. 2 and 3 and Supplementary Movie 3). This observation supports the validity of employing our engram index approach to identify putative engram-containing brain regions with high probability. In addition, many other probable engram carrying regions with high engram index values (e.g., laterodorsal tegmental nucleus or LDTg, cuneiform nucleus, nucleus of the lateral olfactory tract, medial pretectal area, and tuberomammillary nucleus) also exhibited high engram reactivation.

### Optogenetic confirmation of the presence of engrams in some candidate brain regions

**Fig. 4:**
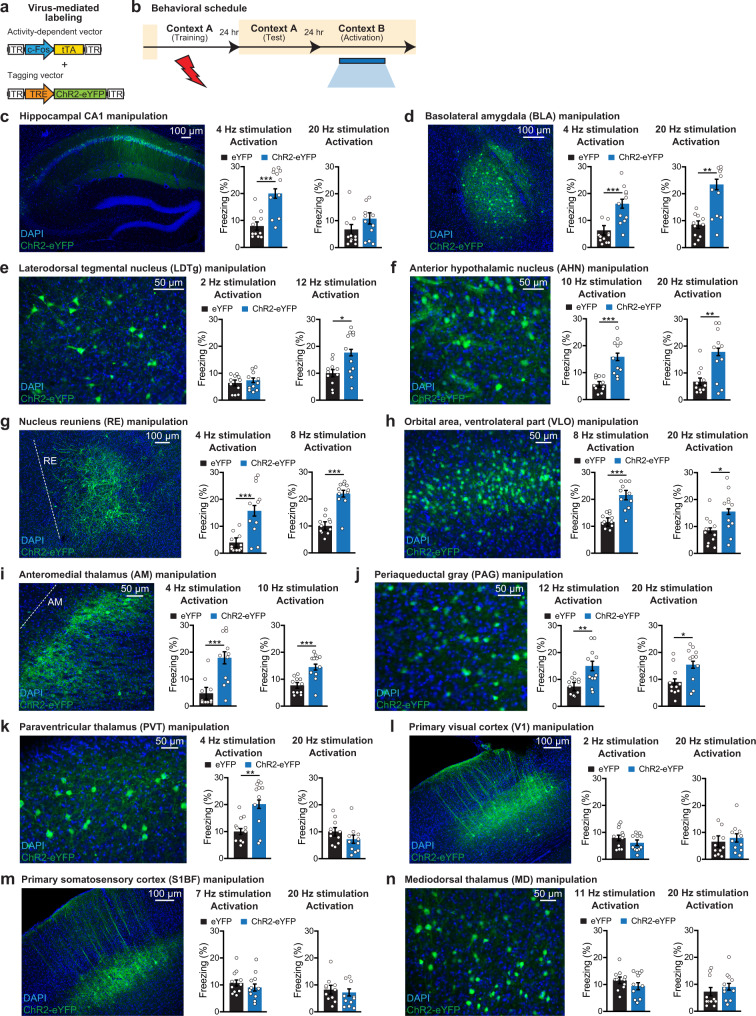
Stimulation frequency-dependent memory retrieval by activating engram cells. **a** cFos^+^ neuron labeling using a cocktail of c-Fos-tTA and TRE-ChR2-eYFP. Wild-type mice raised on doxycycline (DOX) food were injected with the two viruses in the target regions. **b** Behavioral schedule. Beige shading signifies that mice were DOX, precluding ChR2-eYFP expression. Mice were taken off DOX 24 h before CFC. Natural recall test (Test). Optogenetic reactivation session in a neutral context B (Activation). **c** CA1 section (left). eYFP and ChR2-eYFP (*n* = 11 mice per group) groups. **d** BLA section (left). eYFP and ChR2-eYFP (*n* = 11 mice per group) groups. **e** LDTg section (left). eYFP and ChR2-eYFP (*n* = 11 mice per group) groups. **f** AHN section (left). eYFP and ChR2-eYFP (*n* = 11 mice per group) groups. **g** RE section (left). eYFP and ChR2-eYFP (*n* = 11 mice per group) groups. **h** VLO section (left). eYFP and ChR2-eYFP (*n* = 11 mice per group) groups. **i** AM section (left). eYFP and ChR2-eYFP (*n* = 11 mice per group) groups. **j** PAG section (left). eYFP and ChR2-eYFP (*n* = 11 mice per group) groups. **k** PVT section (left). eYFP and ChR2-eYFP (*n* = 11 mice per group) groups. **l** V1 section (left). eYFP and ChR2-eYFP (*n* = 11 mice per group) groups. **m** S1BF section (left). eYFP and ChR2-eYFP (*n* = 11 mice per group) groups. **n** MD section (left). eYFP and ChR2-eYFP (*n* = 11 mice per group) groups. Statistical comparisons are performed using two-tailed unpaired *t*-tests; *P < 0.05, **P < 0.01, ***P < 0.001. Data are presented as mean ± SEM. *P* values: 0.0004 (4 Hz) (**c**), 0.0002 (4 Hz), 0.0011 (20 Hz) (**d**), 0.0105 (12 Hz) (**e**), 9.19E−05 (10 Hz), 0.0071 (20 Hz) (**f**), 0.0006 (4 Hz), 2.56E−06 (8 Hz) (**g**), 1.55E−05 (8 Hz), 0.0214 (20 Hz) (**h**), 0.0006 (4 Hz), 0.0004 (10 Hz) (**i**), 0.0085 (12 Hz), 0.0186 (20 Hz) (**j**), 0.0029 (4 Hz) (**k**). Source data are provided as a Source Data file.

Although the initial demonstration of engrams was made by showing that optogenetic reactivation of hippocampal dentate gyrus engram cells at a frequency of 20 Hz resulted in recall of the specific memory^4^, later studies showed that engram cell ensembles in hippocampal CA1^12^ and prefrontal cortex^10^ are more effectively reactivated using 4 Hz optogenetic stimulation rather than 20 Hz stimulation. These findings indicated that the identification of additional engram cell ensembles by optogenetic recall should be performed using multiple frequencies of laser light. Therefore, in this study we performed optogenetic reactivation at both 4/20 Hz as well as other frequencies that have been identified based on the in vivo firing rates of each brain region. To tag putative engram cells, we employed a double virus approach^7^, which included an activity-dependent vector c-Fos-tTA and a channelrhodopsin-2 (ChR2)-tagging vector TRE-ChR2-eYFP (Fig. 4a). Neurons activated during CFC training were tagged by taking the animals off their doxycycline diet for 24 h before the encoding epoch (Fig. 4b). Following encoding, we performed a natural memory recall test to confirm successful fear memory recall (Supplementary Fig. 2d), after which we reactivated putative engram cells in a neutral context (Fig. 4b).

As a positive control, we observed robust memory recall by the optogenetic reactivation of dorsal CA1 engram cells at 4 Hz specifically (Fig. 4c and Supplementary Fig. 3a), as well as by the reactivation of BLA engram cells at both 4 and 20 Hz frequencies (Fig. 4d and Supplementary Fig. 3b). We then subjected several brain regions to the optogenetic recall tests. These regions had not previously been shown to carry CFC memory engram cell ensembles but the present screening by the engram index displayed significant values (Fig. 2), and also these regions showed high values of engram reactivation induced by recall tests (Fig. 3). Strikingly, all these brain regions induced robust memory recall when they were optogenetically stimulated (Fig. 4e–k and Supplementary Figs. 3c–h, and 4a). The regions are: LDTg, anterior hypothalamic nucleus (AHN), RE thalamus, ventrolateral orbital area (VLO), AM thalamus, periaqueductal gray (PAG), and paraventricular (PVT) thalamus. In general, optogenetic protocols based on the natural firing rates were effective at inducing memory recall. Importantly, using a random labeling approach in these engram-holding brain regions, we could not induce freezing behavior upon optogenetic stimulation of these cell populations (Supplementary Fig. 5). In primary visual area (V1) or primary somatosensory area barrel field (S1BF), the two regions that did not meet the engram index analysis criteria and lacked significant engram reactivation, we were unable to optogenetically induce memory recall (Fig. 4l, m and Supplementary Fig. 3i, j). Similarly, optogenetic reactivation of MD thalamus ensembles did not induce memory recall (Fig. 4n and Supplementary Fig. 3k). This last observation is interesting because MD thalamus showed a low (Fig. 2), but significant engram index value, however it lacked a significant level of reactivation by natural recall cues (Supplementary Table 3). Overall, the application of brain-wide activity mapping by engram index and engram reactivation analyses followed by confirmatory optogenetic recall experiments allowed us to identify additional brain regions that carry engram cells for CFC memory.

### Functional connectivity of an engram ensemble with downstream brain areas

**Fig. 5:**
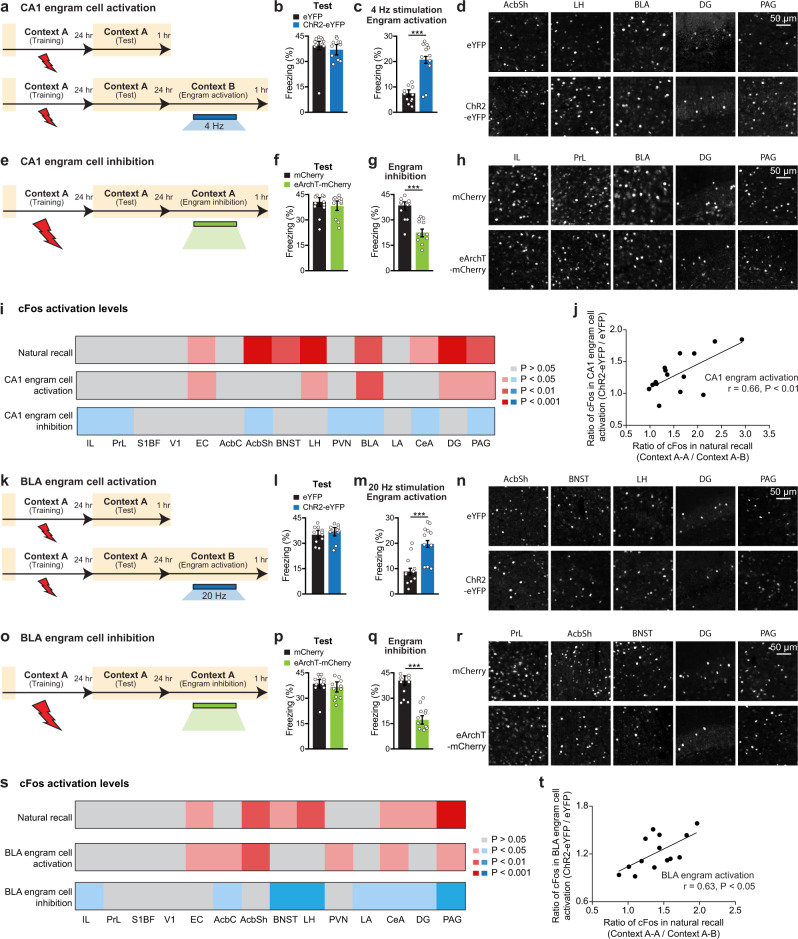
Neuronal activity patterns following engram cell manipulations. **a** Schedule for natural recall (top), engram activation (bottom). **b** Natural memory recall (Test). **c** Optogenetic reactivation. eYFP and ChR2-eYFP (*n* = 11 mice per group) groups. **d** cFos^+^ neurons in representative brain regions, shown for eYFP (top) and ChR2-eYFP (bottom) groups. **e** Schedule for CA1 engram inhibition. **f** Natural memory recall (Test). **g** Optogenetic inhibition. mCherry and eArchT-mCherry (*n* = 11 mice per group) groups. **h** cFos^+^ neurons in representative brain regions, shown for mCherry (top) and eArchT-mCherry (bottom) groups. **i** Heat map represents cFos activation levels in 15 brain regions for natural recall and CA1 engram manipulations (*n* = 7 mice per group). Colored regions indicate an increase/decrease in the number of cFos^+^ neurons based on the *P* value obtained by comparing control vs. manipulation group data. **j** Scatter plot comparing the ratio of cFos^+^ cell counts between CA1 engram activation and natural recall (*n* = 7 mice per group). **k** Schedule for BLA engram activation, as performed in **a**. **l** Natural recall (Test). **m** Optogenetic reactivation. eYFP and ChR2-eYFP (*n* = 11 mice per group) groups. **n** cFos^+^ neurons in representative brain regions, shown for eYFP (top) and ChR2-eYFP (bottom) groups. **o** Schedule for BLA engram inhibition. **p** Natural recall (Test). **q** Optogenetic inhibition. mCherry and eArchT-mCherry (*n* = 11 mice per group) groups. **r** cFos^+^ neurons in representative brain regions, shown for mCherry (top) and eArchT-mCherry (bottom) groups. **s** Heat map represents cFos activation levels in 14 brain regions for natural recall and BLA engram manipulations (*n* = 7 mice per group). **t** Scatter plot comparing the ratio of cFos^+^ cell counts between BLA engram activation and natural recall (*n* = 7 mice per group). Pearson’s correlation coefficient (*r*). Unless specified, statistical comparisons are performed using two-tailed unpaired *t*-tests; ***P < 0.001. Data are presented as mean ± SEM. *P* values: 2.14E−05 (**c**), 0.0002 (**g**), 0.0075 (**j**), 0.0005 (**m**), 1.13E−06 (**q**), 0.0153 (**t**). Source data are provided as a Source Data file. Each experiment was performed in two independent batches that yielded similar results.

Brain-wide mapping of the engram of a specific memory confirms that a given memory is not stored in a single brain region but is stored in a chain of engram cell ensembles dispersed in multiple brain regions. This calls for an experiment that will reveal the functional connectivity of an engram cell ensemble in a brain region with its anatomically downstream areas. In the past, such experiments were conducted successfully in limited cases^11,12^. In the present study, we examined cFos activation in a broader set of downstream brain regions following a putative upstream CFC engram cell reactivation or inhibition. Manual cFos^+^ cell counts from tissue sections were employed for these experiments. In the first set of experiments, we focused on dorsal hippocampal CA1 engram manipulations using the double virus approach. Optogenetic reactivation of CA1 engram cells at 4 Hz resulted in robust memory recall, which was not observed in control eYFP mice (Fig. 5a–c). One hour after optogenetic reactivation of CA1 engram cells in the neutral context, mice were processed for cFos staining. Among 15 brain regions examined, the numbers of cFos-positive cells were significantly increased in EC, lateral hypothalamus (LH), BLA, hippocampal dentate gyrus (DG), and PAG in the CA1 ChR2-eYFP group as compared to the control group in which CA1 engram cells were not optogenetically activated (Fig. 5d, i). To perform a comparable analysis following CA1 engram cell inhibition, we employed a double virus approach expressing eArchT-mCherry in engram cells by injection of an activity-dependent vector c-Fos-tTA and an eArchT tagging vector TRE-eArchT-mCherry. Optogenetic inhibition of CA1 engram cells decreased memory recall in the training context as compared to control mCherry-only mice (Fig. 5e–g). cFos analyses following CA1 engram cell inhibition revealed that prefrontal sub-regions, infralimbic (IL) and prelimbic (PrL) cortices, nucleus accumbens shell (AcbSh), PVN, BLA, central amygdala (CeA), and PAG had significantly decreased neuronal activity as compared to the control group (Fig. 5h, i).

In the second set of experiments, we focused on optogenetic manipulations of BLA engram cells also using the double virus approach. As expected, BLA engram cell reactivation induced memory recall (Fig. 5k–m), and BLA engram cell inhibition decreased memory retrieval in the training context (Fig. 5o–q). cFos analyses following optogenetic activation of BLA engram cells (Fig. 5n, s) showed increased activation in EC, nucleus accumbens core (AcbC), AcbSh, PVN, CeA, and PAG. Conversely, cFos analyses following BLA engram cell inhibition (Fig. 5r, s) showed significantly decreased activity in IL, AcbC, bed nucleus of the stria terminalis (BNST), LH, lateral amygdala (LA), CeA, DG, and PAG regions. These results revealed not only the specific brain regions among those analyzed but also their neural activity pattern changes following upstream engram cell ensemble manipulations.

Different from the engram manipulation observations, cFos activation in random labeling mice showed a significant increase in DG activation for the CA1 random control activation group, and a significant decrease in IL, PrL, and PVN activation for the CA1 random control inhibition group (Supplementary Fig. 6a). These data indicate that a significant increase in EC, LH, BLA, and PAG activation is unique to the CA1 engram cell manipulated group, and that a significant decrease in AcbSh, BLA, CeA, and PAG activation is unique to the CA1 engram cell inhibited group. Following a similar approach for BLA (Supplementary Fig. 6a), we noted a significant increase in AcbC and PVN activation for the BLA random control activation group, and a significant decrease in IL, AcbC, LA, and DG activation for the BLA random control inhibition group. These data indicate that a significant increase in EC, AcbSh, CeA, and PAG activation is unique to the BLA engram cell manipulated group, and that a significant decrease in BNST, LH, CeA, and PAG activation is unique to the BLA engram cell inhibited group. These random control experiments helped identify the unique contribution of engram cells with regards to functional connectivity changes.

A few brain regions (EC and PAG) that are significantly activated by CA1 engram activation are also activated by BLA engram activation. This is also the case for engram inhibition: IL, CeA, and PAG are decreased in both CA1 and BLA inhibition groups. These data suggest that there is a core set of regions that are similarly modulated by CA1 or BLA engram cell manipulations. Based on the hub and spoke hypothesis, certain brain regions (“hubs”) exert greater influence on memory function. It is possible that EC, PAG, IL, and CeA serve as engram hubs because these three regions were modulated by different (CA1 or BLA) engram activation or inhibition manipulations.

### Optogenetic manipulation mimics natural recall cue-driven effects in the engram ensemble pathway

The cFos analyses following optogenetic manipulations of engram cells allowed us to examine individual brain region changes in neuronal activity. Taking advantage of this data set, we next examined the pattern of neuronal activity induced by optogenetic manipulations of engram cells compared to natural memory recall activity patterns more directly. For this purpose, we plotted cFos activity levels for natural recall, ChR2-based engram reactivation groups, and eArchT-based engram inhibition groups (Fig. 5i, s and Supplementary Fig. 6a), which were normalized by their respective control group data. Engram cell reactivation in CA1 showed that memory retrieval is accompanied by enhanced cFos activation in several brain regions that are activated by natural memory recall (Fig. 5i), which was a subset of the natural recall activity pattern. A similar observation was made for engram cell reactivation in BLA (Fig. 5s). Importantly, when we statistically compared the neuronal activity pattern following optogenetic engram cell reactivation in CA1 (Fig. 5j) and BLA (Fig. 5t) to the neuronal activation pattern induced by natural recall, we observed a significant correlation between engram cell reactivation and natural recall for both brain region manipulations. These experiments demonstrate a comparable activity pattern between natural memory recall and optogenetic engram cell reactivation, which suggests that optogenetic reactivation of engram cells recreates at least part of the activity pattern corresponding to natural memory recall. Further, engram cell inhibition in CA1 (Fig. 5i) and BLA (Fig. 5s) showed that memory retrieval impairments are accompanied by decreased cFos activation in several brain regions that are activated by natural memory recall, which is consistent with the observed behavioral disruptions. These data support the idea that memory engram ensembles that are activated by learning are reactivated during recall of the specific memory^1,2^, and that this reactivation is a critical component of successful memory retrieval (Supplementary Fig. 6b–e).

### Simultaneous chemogenetic reactivation of multiple engram cell ensembles in the engram ensemble pathway enhances memory recall

**Fig. 6:**
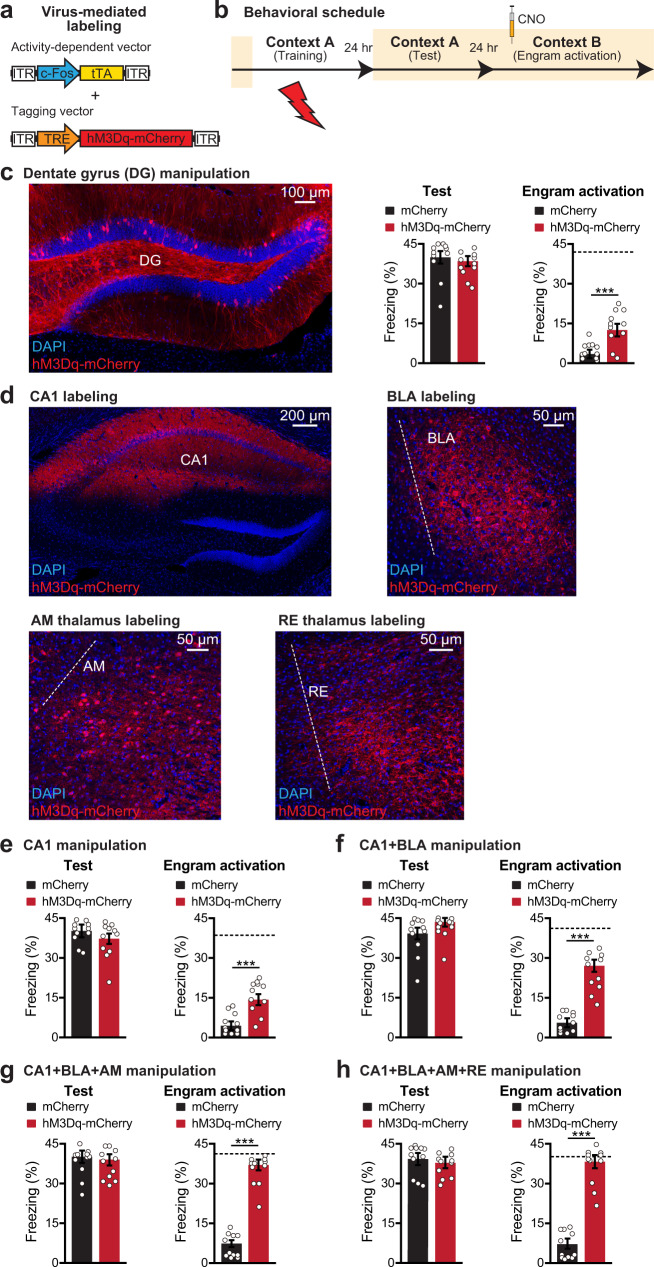
Simultaneous reactivation of multiple engram cell ensembles. **a** Labeling strategy using c-Fos-tTA and TRE-hM3Dq-mCherry in wild-type mice. **b** Behavioral schedule. One day after training, a natural recall test was performed (Test). The next day, mice received CNO intraperitoneally 45 min before a chemogenetic reactivation (Engram activation) session in a neutral context B. **c** Hippocampal DG section showing cFos^+^ neurons labeled with hM3Dq-mCherry. Natural memory recall (Test). Chemogenetic reactivation (Engram activation). mCherry and hM3Dq-mCherry (*n* = 11 mice per group) groups. **d** Hippocampal CA1, BLA, AM thalamus, and RE thalamus sections showing cFos^+^ neurons labeled with hM3Dq-mCherry. **e** Chemogenetic manipulation of CA1 engram cells. Natural recall (Test). Chemogenetic reactivation (Engram activation). mCherry and hM3Dq-mCherry (*n* = 11 mice per group) groups. **f** Chemogenetic manipulation of CA1 and BLA engram cells. Natural recall (Test). Chemogenetic reactivation (Engram activation). mCherry and hM3Dq-mCherry (*n* = 11 mice per group) groups. **g** Chemogenetic manipulation of CA1, BLA, and AM engram cells. Natural recall (Test). Chemogenetic reactivation (Engram activation). mCherry and hM3Dq-mCherry (*n* = 11 mice per group) groups. **h** Chemogenetic manipulation of CA1, BLA, AM, and RE engram cells. Natural recall (Test). Chemogenetic reactivation (Engram activation). mCherry and hM3Dq-mCherry (*n* = 11 mice per group) groups. Dashed line indicates natural recall freezing level (**c**, **e**–**h**). One-way ANOVA followed by Tukey multiple comparison post-hoc tests revealed significant differences between one ensemble (CA1) vs. two ensemble (CA1 and BLA) reactivations (*P* < 0.05), and two ensemble (CA1 and BLA) vs. three ensemble (CA1, BLA, and AM) reactivations (*P* < 0.05). Three ensemble (CA1, BLA, and AM) vs. four ensemble (CA1, BLA, AM, and RE) reactivations were not significantly different. Reactivation of additional engram cell ensembles correlates with enhanced memory reactivation. Unless specified, statistical comparisons are performed using two-tailed unpaired *t*-tests; ***P < 0.001. Data are presented as mean ± SEM. *P* values for activation sessions: 0.0002 (**c**), 6.95E−05 (**e**), 5.87E−07 (**f**), 8.75E−11 (**g**), 9.28E−10 (**h**). Source data are provided as a Source Data file. Each experiment was performed in two independent batches that yielded similar results.

Brain-wide activity mapping using engram index and engram reactivation (Figs. 1–3), memory recall induced by optogenetic engram cell reactivation (Fig. 4), and similar neuronal activation patterns induced by natural memory recall and optogenetic reactivation-based recall (Fig. 5), suggest that multiple engram cell ensembles distributed across the brain contribute to the efficient and specific natural memory retrieval process. Although the optogenetic reactivation of an individual engram cell ensemble results in significant levels of memory recall^4^, the reactivation under naturalistic conditions would involve multiple engram cell ensembles. Indeed, optogenetic reactivation of a single engram cell ensemble does not reach the level of recall that is attained by natural recall^4^. To investigate this issue, we took advantage of chemogenetic neuronal activation using the excitatory hM3Dq DREADDs receptor^24^. This particular approach allowed us to simultaneously activate multiple hM3Dq-expressing engram populations using the ligand clozapine-N-oxide (CNO). We developed a double virus approach expressing hM3Dq-mCherry in engram cells (Fig. 6a). Our behavioral schedule included engram cell labeling during CFC training, followed by a natural memory recall test, and then finally CNO-induced engram cell reactivation in a neutral context on day 3 (Fig. 6b). Because the initial demonstration of engram cell reactivation-induced memory recall was performed in hippocampal DG^4^, we attempted engram cell reactivation using the DREADDs system in DG. Activating DG engram cells using CNO resulted in increased freezing behavior compared to mCherry control mice (Fig. 6c), which validated this chemogenetic approach for engram cell reactivation.

We next performed stepwise multiple engram cell ensemble reactivation experiments. Building on our findings using optogenetic engram cell reactivation (Fig. 4), we reactivated a single engram cell ensemble (CA1), two engram cell ensembles (CA1 and BLA) tagged in the same animals, three engram cell ensembles (CA1, BLA, and AM) tagged in the same animals, and four engram cell ensembles (CA1, BLA, AM, and RE) tagged in the same animals (Fig. 6d). Consistent with the corresponding optogenetic reactivation experiment (Fig. 4c), CNO-induced CA1 engram cell reactivation resulted in memory recall (Fig. 6e). Reactivation of two (Fig. 6f and Supplementary Fig. 4b) and three (Fig. 6g) engram cell ensembles conferred enhanced memory reactivation as assessed by the percentage of time freezing. This finding indicates that the simultaneous reactivation of multiple engram cell ensembles results in greater memory reactivation than their subsets. Interestingly, we did not observe further enhancement of memory reactivation by the simultaneous reactivation of four engram cell ensembles (Fig. 6h and Supplementary Fig. 4b), suggesting that there may be an upper limit in the capacity of memory engram cell reactivation to modulate behavioral outputs. Nevertheless, since the simultaneous reactivation of three and four engram cell ensembles resulted in memory recall strength that was comparable to natural memory recall, this may reflect a mechanism by which the brain adjusts the strength of memory recall depending on the importance of the memory for current and future decisions.

## Discussion

### A four-step approach for brain-wide mapping of memory engram cell ensembles

We performed brain-wide high-throughput screening of putative engram cell ensembles of a contextual fear memory by combining SHIELD-based brain phenotyping technology^19^, the engram index analyses, and the overlap analysis of neurons activated by learning and reactivated by recall. Preservation of both protein fluorescence and tissue architecture by SHIELD enabled automated 3D analysis of fluorescent protein-tagged mouse brains and the generation of brain-wide activity maps of 247 brain regions.

The engram index is based on the concept that engrams are held by neuronal ensembles that are activated by learning and are reactivated to support recall^1–3^. In other words, neurons in a brain region containing engram cells of a specific memory should show high levels of activation during both memory encoding and recall, whereas this region will show lower levels of activation in the home cage. To reflect these criteria, the engram index equation has two components. The first component (i.e., numerator of the equation) computes the increase in the average number of cFos-activated neurons for a given brain region during memory encoding as compared to home cage. The second component (i.e., denominator of the equation) computes the difference between activation levels during memory encoding and recall. In doing so, this component decreases the engram index for brain regions with high activation levels during either encoding or recall, which also helps exclude regions that are primarily responsive to the foot shocks themselves (i.e., false positive candidate engram regions). To summarize, “significant brain regions” with engram indices indicate that these structures satisfy two criteria: (a) a clear increase in activation levels specifically during context-fear associations as compared to home cage experiences, and (b) high similarity between encoding and recall activation levels, which is in agreement with the concept that engram cells are activated during learning and reactivated during recall.

**Fig. 7:**
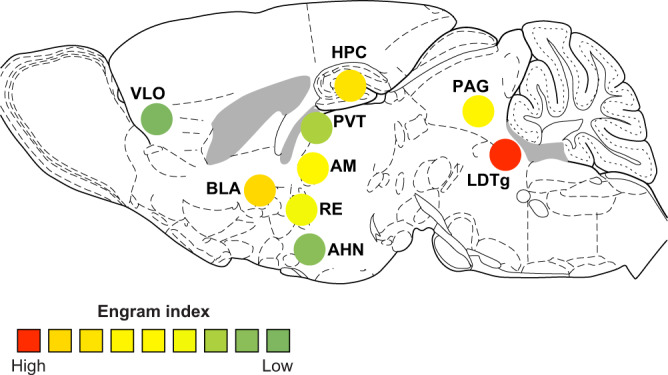
A partial list of constituent engram ensembles in the unified engram complex for contextual fear conditioning memory. Distribution of the confirmed engram cells (from Fig. 4) in a sagittal mouse brain section, where the engram index values correspond to those in Fig. 2. Hippocampus (HPC).

Most known brain regions that have previously been demonstrated to hold engrams by optogenetic and other manipulations (e.g., hippocampal DG, CA3, and BLA) were among the significant brain regions list with engram index values, supporting our hypothesis that a “significant brain region” with an index value is likely to hold an engram cell ensemble. One limitation of this index, however, is that it cannot be used to identify silent engram cells, which are formed in certain brain regions during encoding but are not reactivated by natural recall cues for recall although they can be optogenetically reactivated^11,12,25^. A second limitation of this index is that it is limited to brain regions in which cFos is expressed, which clearly is the case for the majority of regions. However, the majority of “significant brain regions” that gave engram indices were also those that showed significant engram reactivation, making them likely regions to carry engrams and thereby were selected for the cumbersome but powerful functional intervention experiments to confirm the existence of engrams. This permitted us to identify additional brain regions as holders of engram cell ensembles, specifically brainstem (i.e., LDTg), hypothalamus (i.e., AHN), thalamus (i.e., RE, AM, and PVT), cortex (i.e., VLO), and midbrain (i.e., PAG) (Figs. 4 and 7 and Supplementary Fig. 7), none of which had been demonstrated to carry CFC memory engrams prior to this study.

Interestingly, our brain-wide engram mapping experiments revealed that certain brain regions exhibit higher recall activation as compared to CFC memory encoding (e.g., Ol, LH, and AV). We interpret these preferential recall brain regions as those that contain engram cells for CFC, and the activation by recall cues is enhanced in these regions to specifically support recall processes. A previously reported example of a brain region in the literature that is crucial for memory recall and shows enhanced neuronal activity (i.e., cFos) during recall is dorsal subiculum^26^. At present, we do not know the mechanism underlying this recall enhancing process, but one possibility may be the presence of local recurrent circuitry in such brain regions. If the recurrent circuitry is strengthened during the post-encoding time window, subsequent reactivation of engram cells by recall cues may result in firing of connected cells in the region. In this scenario, these brain regions will show greater numbers of cFos activated neurons by recall as compared to CFC memory encoding. In the future, it would be interesting to compare the recall-activated brain regions found in this study with a group of mice in which brain-wide activity mapping is performed after recall of a neutral memory (i.e., by excluding foot shocks from the CFC training epoch). Such analysis would identify brain regions that are particularly active during the recall of a fear memory vs. a neutral experience.

A previous brain-wide activity mapping study investigated the effects of foot shocks or cocaine administration and manipulated prefrontal cortex engram cells^16^. Another study examined the neurons activated by fear conditioning in 200 brain regions and manipulated prefrontal cortex engram cells for remote memory^18^. A third study investigated the coactivation of 84 brain regions for remote fear memory expression and found that an inhibition of the coactivation in some of these regions reduced memory expression^17^. Our four-step experimental strategy has permitted a significantly more complete map of the unified engram complex of a specific memory.

### Potential role of the identified engram cell ensembles

Since AM thalamus receives projections from the medial hypothalamic circuit responsible for threat processing, and has reciprocal connectivity with multiple cortical regions, it is likely that AM thalamus engram cells convey cortico-hypothalamic information to hippocampal circuitry for the generation of context-specific, high-valence memories^27^. Similarly, PVT thalamus is thought to control processes such as arousal, stress, emotional memory, and motivation^28^, which makes it an ideal candidate to contribute to the encoding of CFC memories. On the other hand, RE thalamus has been reported to play a crucial role in hippocampal-dependent encoding of contextual memories, particularly contributing to the discrimination of similar environments^29^. Therefore, RE engram cells may enhance the specificity of contextual memory retrieval by regulating the online discrimination ability of the animal. These findings support the growing idea that thalamic ensembles play an active role in physiological/cognitive functions^30,31^, as opposed to the previously thought passive relay role. Along with thalamic engrams, we also identified additional engram cell ensembles in cortical and other subcortical structures. While VLO engram cells may contribute to long-term memory retention^32^, it is likely that midbrain PAG engram cells underlie the execution of freezing behavior^22^. Although it is well-established that PAG can drive freezing behavior, because an activation of randomly-labeled neurons (~20% of PAG) did not induce freezing, it is possible that there is an underappreciated functional heterogeneity in PAG wherein some ensembles do not contribute to freezing behaviors. Similarly, LDTg engram cells may promote emotional arousal under adverse conditions^33^, and AHN engram cells may regulate the expression of conditioned fear behaviors^34^. In addition to complementary functional contributions, these distributed engram cell ensembles also showed differences with regards to local fiber labeling. Specifically, two thalamic regions (RE and AM) had strongly labeled local fibers, which may indicate connectivity with neighboring neurons in addition to long-range projections. These regions may use different mechanisms for engram cell reactivation as compared to other regions that lacked local fiber labeling (e.g., LDTg, VLO, and PAG).

### Memory storage in distributed engram cell ensembles

The concept that a memory is stored not just in a single engram cell ensemble but in learning-induced enduring changes in multiple functionally connected neuronal ensembles was suggested by Richard Semon (“unified engram complex”)^1^ and Donald Hebb (“neurons that fire together wire together”)^35^. The experimental evidence for this concept came from an observation that gene expression is altered by experience in widespread, behaviorally-defined neural circuits^36^, multiunit recording experiments that identified distributed brain regions involved in memory formation^37^, and an analysis of engram cells from multiple hippocampal subfields and the amygdala^8,12,38^, which has since been supported by activity mapping studies^15–18^. The present study provides three additional types of support for the unified engram complex hypothesis. First, we have revealed the hitherto most comprehensive set of brain regions where the component engrams are localized for a single engram complex. Second, we have shown that optogenetic activation of a single engram cell ensemble (the one in CA1 or BLA) can activate a set of other engram ensembles or candidate engram ensembles, demonstrating functional connectivity of engram ensembles across multiple brain regions. Third, in the literature, optogenetic reactivation of a single engram ensemble, albeit resulted in memory recall, its level never reached that attained by natural recall cues^4,9,10^. On the other hand, Semon’s hypothesis^1^ posits that engram cell ensembles of a specific memory is scattered in multiple brain regions and they would be reactivated simultaneously by natural recall cues. In the present study, we resolved the apparent discrepancy of Semon’s model and optogenetic memory recall by demonstrating that a simultaneous chemogenetic reactivation of multiple component engram ensembles can result in the natural level of recall.

The distributed nature of engram cell ensembles of a specific memory has led to the suggestion that the memory engram within an individual brain region may contribute a subset of the overall memory information^25,39^. For example, hippocampal engrams are thought to primarily contribute contextual information by acting as an index for cortical memories of various sensory modalities^40,41^, whereas amygdala engrams hold valence information for a given experience^8,22,42,43^. In addition, cortical engrams such as those in the retrosplenial and prefrontal cortices may support spatial navigation^44^ and top-down control of memory retrieval^45^, respectively. Further, engrams in auditory^46^ and olfactory cortices^47^ may support auditory recognition memory and odor-induced learned behaviors, respectively.

In conclusion, this study provides evidence supporting the concept that a memory is stored in a functionally connected engram ensembles’ complex distributed broadly across the brain, consistent with Semon’s unified engram complex hypothesis^1^. Despite some caveats, our four-step approach has provided to-date the most comprehensive mapping of engrams- and high probability engram-holding brain regions. Future studies can take advantage of this resource to generate a more extensive map of engram cell ensembles including the identification of their functional connectivity as well as the mnemonic functions of individual ensembles.

## Methods

### Subjects

#### Mice

The C57BL/6J wild type male mice were obtained from Jackson Laboratory. For brain-wide neural activity labeling based on the *c-fos* promoter, we used the previously described c-fos-Cre^ERT2^ mouse line^20^. These mice are also known as Fos^CreER^ or FosTRAP mice in which cFos-positive neurons can be labeled by the intraperitoneal injection of 4-hydroxytamoxifen (4-OHT) within a user-defined time-window. For our brain-wide labeling experiments, FosTRAP mice were crossed with the Cre-dependent tdTomato reporter mouse line Ai14, which were obtained from Jackson Laboratory (Stock No. 007908). All mouse lines were maintained as hemizygotes. Mice had access to food and water ad libitum and were socially housed in numbers of two to five littermates until surgery. Following surgery, mice were singly housed. For labeling and behavioral experiments, all mice were male and 3–5 months old. For virus-mediated activity-dependent labeling experiments^7^, wild type male mice had been raised on food containing 40 mg kg^−1^ doxycycline (DOX) for at least one week before surgery and remained on DOX for the remainder of the experiments except for 24 h preceding the target-labeling day. For brain-wide activity dependent labeling followed by SHIELD tissue clearing and brain-wide cFos staining, male mice were 3–6 months old at the time of 4-OHT labeling. All animals were housed in a 7 am–7 pm light on-off cycle facility with a temperature of 18–23 °C and humidity maintained between 40–60%. All experiments were conducted in accordance with U.S. National Institutes of Health (NIH) guidelines and the Massachusetts Institute of Technology Department of Comparative Medicine and Committee of Animal Care.

### Brain-wide activity-dependent labeling

FosTRAP mice crossed to Ai14 reporter mice were employed. 4-OHT (Sigma-Aldrich) was dissolved in 100% ethanol solution by shaking at 37 °C for 20–30 min. One-part castor oil to four parts sunflower oil was combined to prepare the oil mixture that would eventually be injected intraperitoneally (IP). Dissolved 4-OHT was combined with the oil mixture, followed by ethanol evaporation using a vacuum centrifuge. The final concentration of 4-OHT dissolved in the oil mixture was 10 mg ml^−1^. For each mouse, optimal activity-dependent labeling was achieved using a target concentration of 30–40 mg kg^−1^. One hour prior to the behavioral epoch of interest, mice were injected with 4-OHT. Following behavior experiments, mice were returned to their home cages and remained undisturbed for at least 72 h.

### SHIELD processing and clearing

For SHIELD tissue processing^19^, mice were perfused first with ice-cold 1× PBS solution, followed by ice-cold SHIELD perfusion solution (4% w/v paraformaldehyde with the supernatant of 10% w/v polyglycerol 3-polyglycidyl ether (P3PE) resin prepared in 1× PBS). P3PE resin was obtained from EPM-CVC Thermoset Specialties. After 2 days of incubation in the perfusion solution at 4 °C, each brain sample was split into two hemispheres and further incubated in the supernatant of 10% w/v P3PE resin prepared in 1× PBS at 4 °C for 24 h. After 24 h of subsequent incubation in 0.1 M carbonate buffer solution (pH 10.0) at 37 °C, brain hemispheres were transferred to 1× PBS solution containing 0.02% sodium azide and were stored until the delipidation process. For delipidation, brain hemispheres were incubated in a solution containing 10 mM sodium borate, 100 mM sodium sulfite, and 300 mM sodium dodecyl sulfate (pH 9.0 using sodium hydroxide) at 37 °C for 1 day, followed by 8–10 days of incubation at 45 °C with shaking. Once the brain hemispheres were rendered evenly translucent based on visual inspection, they were incubated in iohexol-based PROTOS solution (125 g iohexol, 3 g diatrizoic acid, and 5 g N-methyl-d-glucamine in 110 ml distilled water with a final refractive index set to 1.46) for over 24 h at room temperature for optical clearing.

### Whole brain cFos immunostaining

Volumetric immunolabeling was carried out using the 3D ultrafast immunostaining technique termed eFLASH^23^. Briefly, 350 ml of the main buffer was loaded into the staining device and 5 ml of the sample buffer was loaded into the sample cup. The tissue sample was placed in a nylon mesh then placed into the sample cup. Primary antibody targeting cFos protein (ab214672, Abcam, 1:200 dilution) was added in the sample cup. After 20 h of running the machine (90 V with 500 mA maximum current, temperature set to 25 °C, sample cup stir bar rotation was set to 850 rpm, and sample cup rotation speed was set to 0.01 rpm), the sample buffer was replaced with 5 ml of the fresh sample buffer with secondary antibodies. Dye-conjugated Fc-specific Fab fragments were used for secondary antibody staining (1:400 dilution, Jackson ImmunoResearch). After eight additional hours of running of the machine, the sample was washed in PBS with 0.02% sodium azide at room temperature overnight, and fixed in 4% paraformaldehyde solution in PBS at room temperature for 12 h. After washing the sample in fresh PBS with 0.02% sodium azide, the sample was optically cleared and imaged using light-sheet microscopy.

### Light-sheet microscopy

Transparent brain hemispheres were secured onto a sample holder using 1.5% agarose prepared in the PROTOS solution. After the tissue-agarose mold was fully equilibrated in the PROTOS solution (no visible haze at the tissue-agarose interface and the agarose-solution interface), individual brain hemispheres were imaged using a custom-built light-sheet microscope. Brain-wide tdTomato signal detection (excited by a 561 nm laser) and autofluorescence signal detection (excited by a 635 nm laser) was performed using a 10×/0.6NA objective lens. For brain-wide cFos signal detection, an excitation wavelength of 488 nm was used. After image acquisition, datasets were 4× down-sampled in the *xy* plane to obtain 2.34 × 2.34 × 5 µm (*x*, *y*, *z*) voxel size. Illumination correction was performed with a custom-generated MATLAB script, and images were stitched using TeraStitcher.

### Quantitative activity mapping

Brain hemisphere autofluorescence images were down-sampled and automatically aligned to the annotated autofluorescence atlas from the Allen Brain Institute (version 3)^48^ by linear and non-linear image transformation processes. After manually validating alignment accuracy for individual samples, tdTomato images were projected in order to perform spot detection by local maxima and watershed transformation. To detect tdTomato^+^ neurons, curvature and intensity information are used to select candidate center coordinates. Centered by the candidate coordinates, 16 × 16 × 8 px (*x*, *y*, *z*) patches were cropped and served as training data for a deep learning-based model to finalize the detection. As the voxel size of the image data is 1.8 × 1.8 × 2.0 µm (*x*, *y*, *z*), a patch covers 28.8 × 28.8 × 16 µm area. In total, 34,000 training and 16,300 test patches were generated. The model is trained to classify whether the chosen area contains a neuron or not. This process allowed us to filter false positive center coordinates from the candidate dataset. We designed a ResNet^49^ backboned 3D convolutional neural network for our volumetric data to increase both training and inference speed. The training took 12 h and achieved 90 ± 4% accuracy (from multiple models using different training parameter sets). Detection accuracy of individual hemisphere datasets was confirmed by manual inspection. Detected spot information combined with atlas alignment data enabled the quantification of brain region-specific tdTomato^+^ cell counts. Activated neuron counts were obtained from 247 individual brain regions from each hemisphere except the medulla, because the discrepancy between autofluorescence from this structure in cleared brain hemispheres vs. version 3 of the Allen Brain reference atlas (generated from PFA-fixed brain sections) interfered with automatic region segmentation of this structure. We also excluded fiber tracts since the number of activated neurons in these structures was negligible.

Figure 1b shows a heat map of several major brain regions from the brain-wide activity mapping experiments. The cFos^+^ (i.e., tdTomato^+^) cell counts in this figure panel are from contextual fear conditioning (CFC) and recall (RE) behavioral groups, which were normalized to counts from the home cage (HC) group. Supplementary Tables 1 and 2 lists all brain regions and their subdivisions that were analyzed in these experiments. Accessory supraoptic group was the only region that did not contain activated neurons across behavioral conditions.

Before calculating engram indices, we first identified brain regions in which the mean activated neuronal counts of the CFC and RE groups were individually significantly greater than HC counts (using a one-way ANOVA followed by Tukey post-hoc test). We referred to these areas as “significant brain regions” (Supplementary Table 1), for which we calculated the *engram index* using the following equation:

$${{{{{\mathrm{Engram}}}}}}\, {{{{{\mathrm{index}}}}}}={{{{{{\mathrm{log }}}}}}}_{10}\left(\,\frac{{{|}}{\bar{\mu }}_{{{{{{{\mathrm{CFC}}}}}}}}-{\bar{\mu }}_{{{{{{{\mathrm{HC}}}}}}}}{{|}}}{{{|}}{\bar{\mu }}_{{{{{{{\mathrm{CFC}}}}}}}}-{\bar{\mu }}_{{{{{{{\mathrm{RE}}}}}}}}{{|}}}\right),$$

*μ* is the mean number of activated neurons for individual behavioral groups. This equation includes a common logarithm (base 10) for plotting purposes. The absolute numbers for both the numerator and the denominator allowed us to calculate engram indices from brain regions in which counts in Recall were higher than those in CFC (i.e., such regions would result in negative numbers). We calculated engram indices for significant brain regions and rank-ordered them in Fig. 2b, c. Supplementary Table 2 lists the non-significant brain regions along with their mean activated cell counts across groups. The tdTomato^+^cFos^+^ co-positivity (i.e., engram reactivation) was measured by counting tdTomato^+^ neurons whose center coordinate overlaps with high intensity areas of the cFos-staining channel (plotted as a percentage of the tdTomato^+^ neuronal counts). Significant engram reactivation used a one-sample *t*-test against chance level. Chance level was calculated relative to NeuN staining (1:500 dilution, catalog no. MAB377, Millipore) for each region using the number of recall-activated cFos^+^ neurons. The threshold is set to 0.6 for determining high intensity from an intensity probability map. Imaris (version 9.5) software was used for 3D rendering of tdTomato hemisphere images.

### Viral constructs

To label memory engram cells in wild type mice maintained on DOX food, we used a double-virus system that combined the c-Fos-tTA virus with a TRE-dependent virus. The pAAV-c-Fos-tTA plasmid was previously reported^7^. Similarly, the following TRE-dependent constructs were also previously reported^4,12^: pAAV-TRE-ChR2-eYFP, pAAV-TRE-eYFP, and pAAV-TRE-mCherry. The pAAV-TRE-Cre plasmid was obtained from Addgene (catalog no. 85040). The pAAV-TRE-eArchT-mCherry and pAAV-TRE-hM3Dq-mCherry plasmids were constructed by introducing the eArchT and hM3Dq fragments, respectively, into the pAAV-TRE-mCherry plasmid backbone. The pAAV-DIO-eArchT-mCherry plasmid was constructed by replacing the TRE fragment with a Syn-DIO fragment in the pAAV-TRE-eArchT-mCherry plasmid backbone. The pAAV-TRE-hM4Di-mCherry plasmid was constructed by introducing the hM4Di fragment into the pAAV-TRE-mCherry plasmid backbone. These AAV vectors were serotyped with AAV_9_ coat proteins and packaged at the University of Massachusetts Medical School Gene Therapy Center and Vector Core or Boston Children’s Hospital Viral Core. The AAV_9_-DIO-ChR2-eYFP virus was purchased from Addgene (catalog no. 20298-AAV9). Viral titers were 1.5 × 10^13^ genome copy (GC) ml^−1^ for AAV_9_-c-Fos-tTA, AAV_9_-TRE-ChR2-eYFP and AAV_9_-TRE-eYFP, 2 × 10^13^ GC ml^−1^ for AAV_9_-TRE-eArchT-mCherry, 3 × 10^13^ GC ml^−1^ for AAV_9_-TRE-mCherry, 1.3 × 10^13^ GC ml^−1^ for AAV_9_-TRE-hM3Dq-mCherry, and 7 × 10^12^ GC ml^−1^ for AAV_9_-TRE-Cre, AAV_9_-DIO-eArchT-mCherry, AAV_9_-TRE-hM4Di-mCherry and AAV_9_-DIO-ChR2-eYFP.

### Surgery and optic fiber implants

Mice were anesthetized with isoflurane or 500 mg kg^−1^ avertin for stereotaxic injections. Injections were targeted bilaterally to V1 (−2.7 mm AP, +/−2.5 mm ML, −1.1 mm DV), S1BF (−1.58 mm AP, +/−2.75 mm ML, −1.5 mm DV), BLA (−1.46 mm AP, +/−3.3 mm ML, −4.68 mm DV), AM (−0.7 mm AP, +/−0.63 mm ML, −3.7 mm DV), CA1 (−2.1 mm AP, +/−1.5 mm ML, −1.4 mm DV), RE (−0.58 mm AP, +/−0.25 mm ML, −4.15 mm DV), LDTg (−4.96 mm AP, +/−0.4 mm ML, −3.25 mm DV), AHN (−0.94 mm AP, +/−0.4 mm ML, −5.0 mm DV), VLO (+2.46 mm AP, +/−1.0 mm ML, −2.7 mm DV), PAG (−3.6 mm AP, +/−0.35 mm ML, −2.6 mm DV), PVT (−1.7 mm AP, +/−0.25 mm ML, −2.95 mm DV), and MD (−1.06 mm AP, +/−0.35 mm ML, −3.0 mm DV). Injection volumes were 300 nl for V1 and S1BF, 200 nl for BLA, LDTg, AHN, VLO, PAG, PVT, and MD, 150 nl for AM and RE, and 375 nl for CA1. Viruses were injected at 70 nl min^−1^ using a glass micropipette attached to a 10 ml Hamilton microsyringe. The needle was lowered to the target site and remained for 5 min before beginning the injection. After the injection, the needle stayed for 10 min before it was withdrawn. Custom implants containing two optic fibers (200 mm core diameter; Doric Lenses) was lowered above the injection site for CA1 (0.15 mm above the injection DV). Single optic fiber implants (200 mm core diameter; Doric Lenses) were lowered above the V1, S1BF, BLA, LDTg, AHN, VLO, PAG, PVT, MD, AM, and RE injection sites (0.15 mm above the injection DV). The implant was secured to the skull with two jewelry screws, adhesive cement (C&B Metabond), and dental cement. An opaque cap derived from the top part of a black Eppendorf tube protected the implant. Mice were given 1.5 mg kg^−1^ metacam as analgesic and allowed to recover for 2 weeks before behavioral experiments. All injection sites were verified histologically. As criteria, we only included mice with virus expression limited to the targeted regions.

### Immunohistochemistry

Mice were dispatched using 750–1000 mg kg^−1^ avertin and transcardially perfused with 1× PBS solution, followed by 4% paraformaldehyde (PFA). Brains were extracted and post fixed in 4% PFA at 4 °C for 24 h. Brains were transferred to 1× PBS and 50 µm coronal slices were prepared using a vibratome. For immunostaining, each slice was placed in PBS + 0.3% Triton X-100 (PBS-T), with 5% normal goat serum for 1 h and then incubated with primary antibody at 4 °C for 24 h. Slices then underwent three wash steps for 10 min each in PBS, followed by a 2 h incubation with secondary antibody at room temperature. After three more wash steps of 10 min each in PBS-T, slices were mounted using VECTASHIELD mounting medium on positively charged glass slides. Antibodies used for staining were as follows: chicken anti-GFP (1:1000 dilution, Life Technologies) and anti-chicken Alexa-488 (1:500 dilution), rabbit anti-RFP (1:1000 dilution, Rockland Inc.) and anti-rabbit Alexa-555 (1:500 dilution), cFos was stained with rabbit anti-cFos (1:400 dilution, Santa Cruz) and anti-rabbit Alexa-555 (1:500 dilution), and nuclei were stained with DAPI (1:3000 dilution, Sigma). For counterstaining in Fig. 5, brain sections were incubated with Neuro Trace Fluorescent Nissl Stain (1:100 dilution, Molecular Probes) in 1× PBS for 1 h after secondary antibody staining.

### Behavior assays

Experiments were conducted during the light cycle (7 am to 7 pm). Mice were randomly assigned to experimental groups for each experiment. Mice were habituated to investigator handling for 1–2 min on three consecutive days. Handling took place in the holding room where the mice were housed. Prior to each handling session, mice were transported by wheeled cart to and from the vicinity of behavior rooms to habituate them to the journey. For natural memory recall sessions, data were quantified using FreezeFrame (version 4) software. Optogenetic manipulations interfered with motion detection, and therefore freezing behavior in these experiments were manually quantified. All behavior experiments were collected and analyzed blind to experimental group. Following behavioral protocols, brain sections were prepared to confirm efficient viral labeling in target areas. Animals lacking adequate labeling were excluded prior to behavior quantification.

### Contextual fear conditioning

Two distinct contexts were employed for the contextual fear-conditioning (CFC) paradigm. The conditioning context were 29 × 25 × 22 cm chambers with grid floors, dim white lighting, and scented with 1% acetic acid. The neutral context consisted of 30 × 25 × 33 cm chambers with white perspex floors, red lighting, and scented with 0.25% benzaldehyde. All mice were conditioned (180 s exploration, one 0.75 mA shock of 2 s duration at 180 s, second 0.75 mA shock of 2 s duration at 240 s, 120 s post-shock period), and natural memory recall tests (3 min) were performed one day later. Experiments showed no generalization in the neutral context. Floors of chambers were cleaned with quatricide before and between runs. Mice were transported to and from the experimental room in their home cages using a wheeled cart. The cart and cages remained in an anteroom to the experimental rooms during all behavioral experiments. For activity-dependent labeling, mice were either injected with 4-OHT 1 h prior to behavior (for FosTRAP experiments) or kept on regular food without DOX for 24 h prior to training (for TRE/tTA experiments using B6 mice). For B6 mice, when training or recall was complete, mice were switched back to food containing 40 mg kg^−1^ DOX.

### Optogenetic manipulations

For light-induced freezing behavior, a context distinct from the CFC training chamber (context A) was used. These were 30 × 25 × 33 cm chambers with perspex floors, square ceilings, white lighting, and scented with 0.25% benzaldehyde. Chamber ceilings were customized to hold a rotary joint (Doric Lenses) connected to two 0.32 m patch cords. All mice had patch cords fitted to the optic fiber implant prior to testing. Two mice were run simultaneously in two identical chambers. ChR2 was stimulated at different frequencies (15 ms pulse width) using a 473 nm laser (10–15 mW), for the designated epochs. Testing sessions were 3 min in duration with light on for the entire duration. At the end of 3 min, the mouse was detached and returned to its home cage. Floors of chambers were cleaned with quatricide before and between runs. For green light inhibition experiments, inhibition was performed during the entire memory recall duration (3 min) using a 561 nm laser (~12 mW, constant green light). The brain regions we focused on for optogenetic validation experiments in this study meet all the following criteria: a) Have an engram index value, which means the region shows significantly increased cFos activation relative to HC not only in CFC, but also in recall; b) Significant engram cell reactivation during recall; c) Can be targeted with high specificity using viral injections into the mouse brain; d) Since we aimed to reveal additional engram ensembles, we deliberately did not focus on brain regions that are part of the hippocampal formation, given that they may be expected to hold engram cells for a CFC memory; and e) We sampled brain regions across the entire range of engram index values, which were distributed across the brain.

### Chemogenetic activation

For chemogenetic experiments, we employed the excitatory DREADDs receptor hM3Dq or the inhibitory DREADDs receptor hM4Di. These receptors are activated by the ligand clozapine-N-oxide (CNO), which is injected intraperitoneally (IP). For activation experiments following CFC training and engram cell labeling, 4 mg kg^−1^ CNO was injected IP 50 min before placing the mice in a context distinct from the CFC training chamber. Freezing behavior was automatically quantified during a 3 min session.

### Statistics and reproducibility

Each cell counting and behavior experiment reported in this study was performed in at least two independent batches that yielded similar results.

### Brain-wide activity mapping data analysis

Data were analyzed using Prism 6 software. For engram index analyses, significant brain regions were identified using a one-way ANOVA followed by Tukey multiple comparison post-hoc tests. For engram reactivation analyses, significant brain regions were identified using a two-tailed one-sample *t*-test against chance level.

### Freezing behavior analysis

Data are presented as mean values accompanied by SEM. No statistical methods were used to predetermine sample sizes. Data analysis was performed blind to the conditions of the experiments. Data were analyzed using Microsoft Excel with the Statplus plug-in and Prism 6 software. Statistical comparisons used two-tailed unpaired *t*-tests, one-way ANOVA followed by Bonferroni multiple comparison post-hoc tests, and one-way ANOVA followed by Tukey multiple comparison post-hoc tests (*P* > 0.05 NS, *P < 0.05, **P < 0.01, ***P < 0.001). Statistical parameters including the exact value of *n*, *P*, precision measures (mean ± SEM), and significance are reported in figures and their legends.

### Manual cFos cell counts following engram cell manipulations

Brain slices containing target brain regions were selected based on Nissl staining compared with a standard brain atlas (Franklin and Paxinos, 3rd edition). These sections were used to count the number of cFos^+^ cells within individual regions (Fig. 5). The number of cFos^+^ cells in a 1 mm^2^ area were counted using ImageJ (version 1.53) and MATLAB software. Data were analyzed using Microsoft Excel and Prism 6 software. Heat maps represent cFos activation levels in natural recall, CA1 or BLA engram cell activation, and CA1 or BLA engram cell inhibition, respectively. Specifically, context A-A (trained context) vs. context A-B (neutral context) for natural recall ratios, ChR2-eYFP vs. eYFP for engram cell activation ratios, and eArchT-mCherry vs. mCherry for engram cell inhibition ratios were used. Red colored heat map regions indicate an increase in the number of cFos^+^ neurons based on the P value obtained by comparing individual control vs. natural recall or manipulation group data, whereas blue colored regions indicate a decrease in the number of cFos^+^ neurons (Fig. 5i, s). These statistical comparisons used unpaired *t*-tests. For scatter plots (Fig. 5j, t), the ratios of cFos^+^ cell counts were obtained by normalization to individual control group data. Each dot on the plot represents the ratio of cFos^+^ cell counts in individual brain regions, and “r” represents the Pearson’s correlation coefficient (using two-tailed tests).

### Reporting summary

Further information on research design is available in the Nature Research Reporting Summary linked to this article.

## Supplementary information

Supplementary Information File

Supplementary Movie 1

Supplementary Movie 2

Supplementary Movie 3

Reporting Summary

## Data Availability

Supplementary Information/Source Data are provided with this paper. Whole brain imaging data is available from the laboratory of Prof. Kwanghun Chung upon reasonable request. Source data are provided with this paper.

## Source data

Source Data

## Code availability

Code is available on GitHub: https://github.com/chunglabmit/bmtrap_2021.
