# Supplementary material for: Brain-wide mapping reveals that engrams for a single memory are distributed across multiple brain regions
Source: Nat Commun. 2022 Apr 4;13:1799. doi: 10.1038/s41467-022-29384-4 (PMC8980018; doi:10.1038/s41467-022-29384-4)
Supplement: Supplementary file 1 — Supplementary Information File [file 41467_2022_29384_MOESM1_ESM.pdf]

Supplementary Information for:

Brain-wide mapping reveals that engrams for a single memory are distributed across multiple brain regions

Dheeraj S. Roy*, Young-Gyun Park*, Minyoung E. Kim*, Ying Zhang*, Sachie K. Ogawa*, Nicholas DiNapoli, Xinyi Gu, Jae H. Cho, Heejin Choi, Lee Kamentsky, Jared Martin, Olivia Mosto, Tomomi Aida, Kwanghun Chung, and Susumu Tonegawa

*These authors contributed equally to this work.

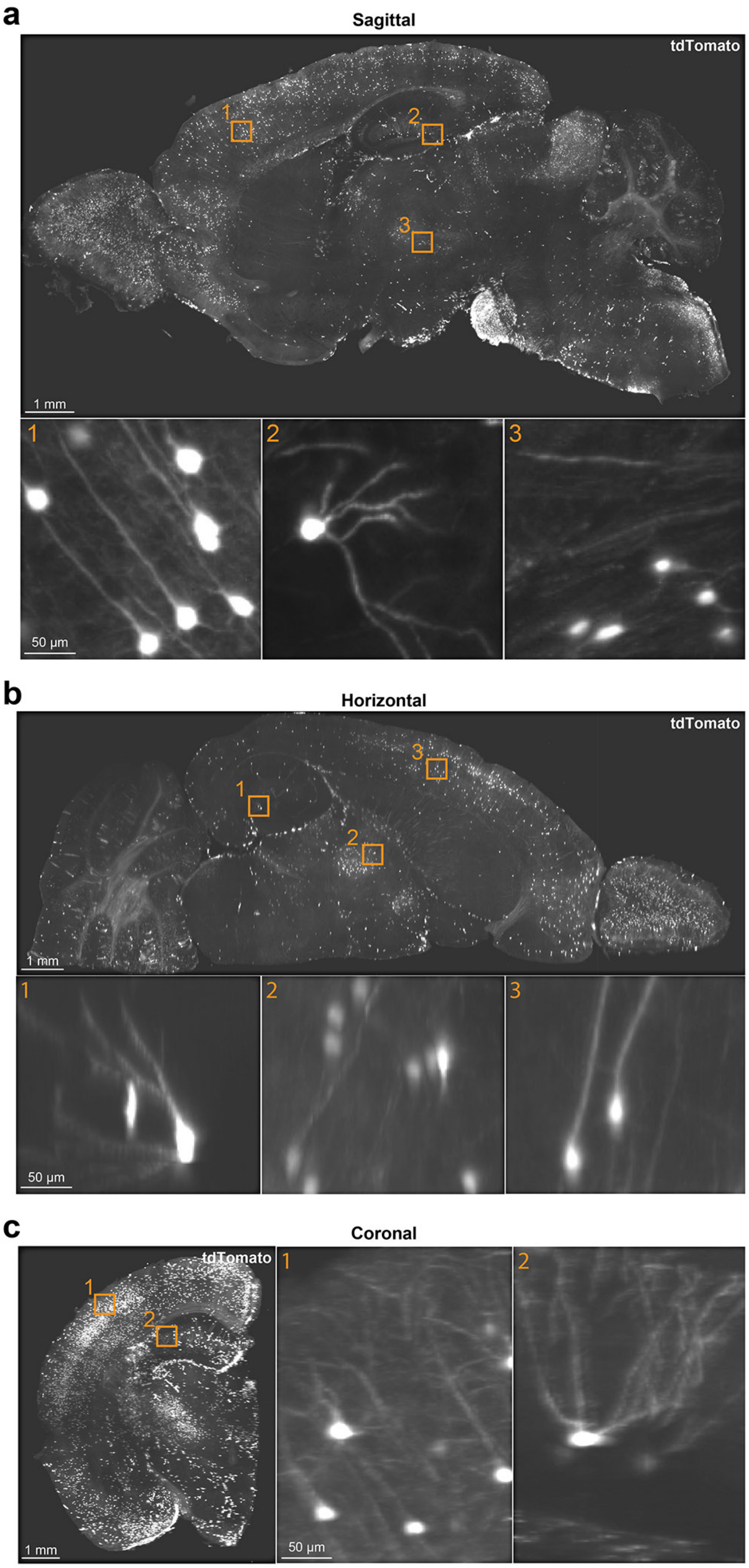
a
Sagittal
tdTomato
1
2
3
1 mm
1
2
3
50 µm
b
Horizontal
tdTomato
3
1
2
1 mm
1
2
3
50 µm
c
Coronal
tdTomato
1
2
1 mm
1
2
50 µm

Supplementary Fig. 1. Orthogonal views of a 3D imaged CFC brain. a-**c**, 3D reconstructions were used to quantify brain-wide $cFos^+$ neuronal ensembles labeled during different behavioral epochs. Using a CFC brain as an example, we extracted 2D orthogonal views from these 3D reconstructions. Sagittal views (**a**), horizontal views (**b**), and coronal views (**c**) demonstrate the single cell resolution achieved using our brain-wide activity mapping approach.

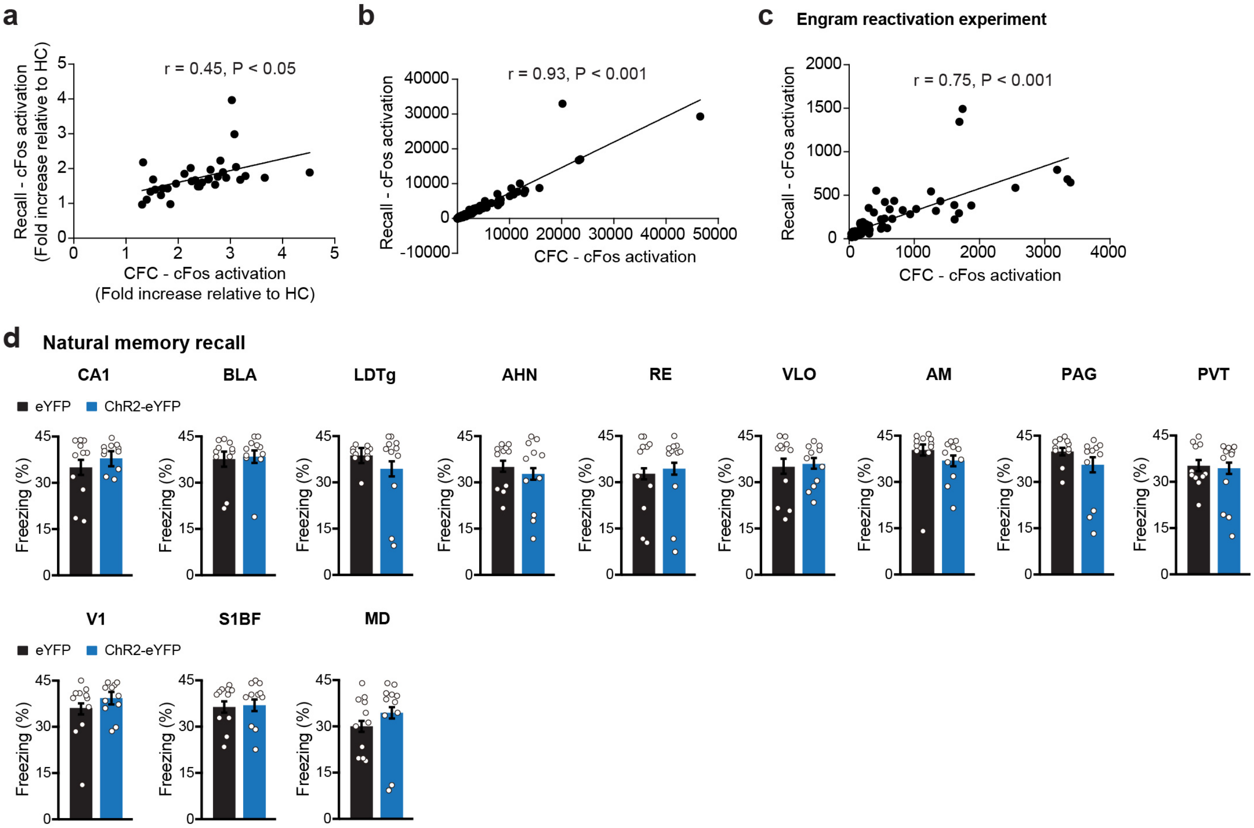

Supplementary Fig. 2. Correlation between cFos activation in CFC and recall epochs, and natural memory recall test data for optogenetic engram reactivation behavioral groups. a-c, Scatter plots between cFos activation in CFC and recall for the subset of regions in Fig. 1b (**a**), for all analyzed regions in Supplementary Tables 1 and 2 using mean $cFos^+$ neuronal counts (**b**), and for the identified significant regions from the engram reactivation experiment in Fig. 3 (**c**). Pearson's correlation coefficient (r). **d**, Recall tests for the nine brain regions that showed optogenetic-induced memory recall in Fig. 4c-k (top row). Recall tests for the three brain regions that did not show optogenetic-induced memory recall in Fig. 4l-n (bottom row) (n = 11 mice per group). Statistical comparisons are performed using two-tailed correlation (**a-c**) and two-tailed unpaired *t* (**d**) tests. Data are presented as mean ± SEM. P values: 0.0071 (**a**), <0.0001 (**b**), <0.0001 (**c**). Source data are provided as a Source Data file.

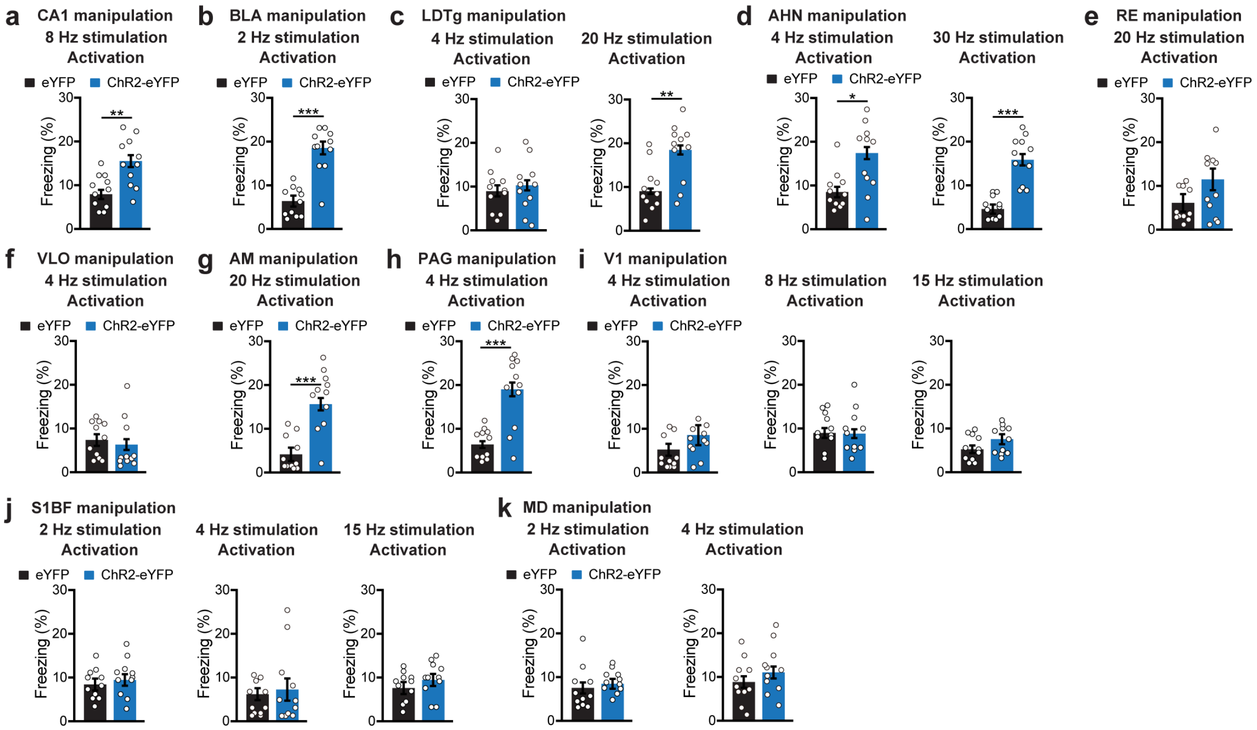

Supplementary Fig. 3. Optogenetic reactivation of CFC-labeled ensembles using additional frequency protocols. a-k, Similar to the experiments in Fig. 4, CA1 groups were tested using a 8 Hz protocol (**a**), BLA groups were tested using a 2 Hz protocol (**b**), LDTg groups were tested using 4 Hz and 20 Hz protocols (**c**), AHN groups were tested using 4 Hz and 30 Hz protocols (**d**), RE groups were tested using a 20 Hz protocol (**e**), VLO groups were tested using a 4 Hz protocol (**f**), AM groups were tested using a 20 Hz protocol (**g**), PAG groups were tested using a 4 Hz protocol (**h**), V1 groups were tested using 4 Hz, 8 Hz, and 15 Hz protocols (**i**), S1BF groups were tested using 2 Hz, 4 Hz, and 15 Hz protocols (**j**), and MD groups were tested using 2 Hz and 4 Hz protocols (**k**). eYFP and ChR2-eYFP (n = 11 mice per group) groups. Statistical comparisons are performed using two-tailed unpaired *t* tests; $*P < 0.05$, $**P < 0.01$, $***P < 0.001$. Data are presented as mean ± SEM. P values: 0.0057 (**a**), 3.99E-06 (**b**), 0.0041 (20 Hz) (**c**), 0.0143 (4 Hz), 5.51E-06 (30 Hz) (**d**), 3.43E-05 (**g**), 0.0001 (**h**). Source data are provided as a Source Data file.

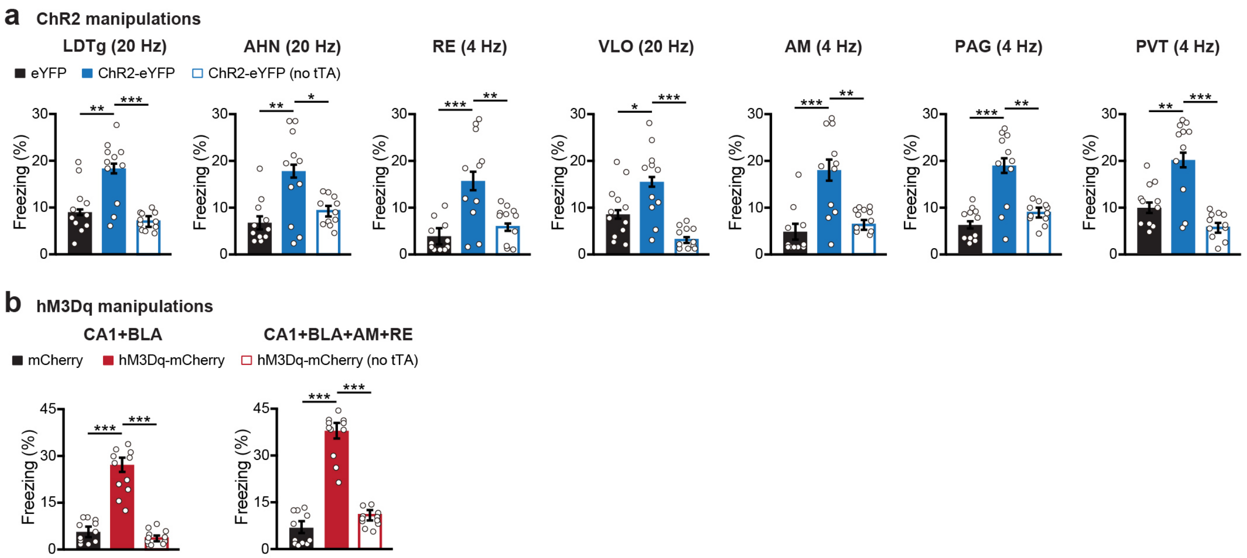

Supplementary Fig. 4. TRE-alone control groups for ChR2 and hM3Dq engram activation experiments. **a**, In Fig. 4, engram cells were optogenetically activated to induce memory recall (solid bars). In this panel, surgery mice were prepared that lacked the c-Fos-tTA component of the viral cocktail (i.e., only received TRE virus injections). These animals are referred to as the "no tTA" group (n = 11 mice per group), which failed to induce memory recall upon light stimulation. **b**, In Fig. 6, CA1+BLA and CA1+BLA+AM+RE engram cells were activated using CNO to induce memory recall (solid bars). In this panel, surgery mice were prepared that lacked the c-Fos-tTA component of the viral cocktail (i.e., only received TRE virus injections). These "no tTA" animals (n = 11 mice per group) failed to induce memory recall upon CNO injections. Statistical comparisons are performed using a one-way ANOVA followed by Bonferroni post-hoc tests; *P < 0.05, **P < 0.01, ***P < 0.001. Data are presented as mean ± SEM. P values for ChR2/hM3Dq vs. no tTA: 3.43E-05 (LDTg), 0.0161 (AHN), 0.0046 (RE), 0.0001 (VLO), 0.0031 (AM), 0.0012 (PAG), 4.03E-05 (PVT) (**a**), 5.30E-08 (CA1+BLA), 1.54E-09 (CA1+BLA+AM+RE) (**b**). Source data are provided as a Source Data file.

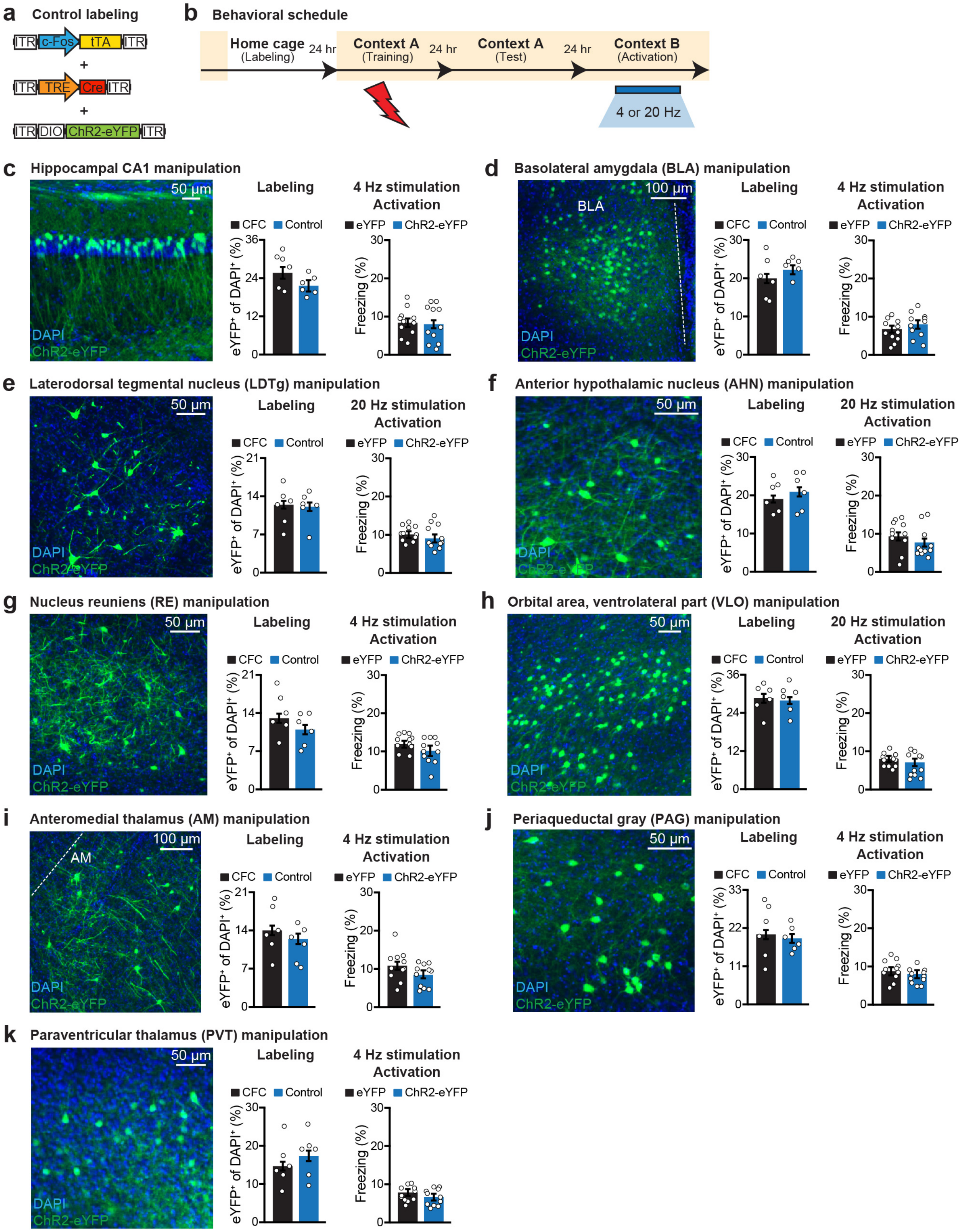

Supplementary Fig. 5. Activation of randomly labeled cell populations in individual engram brain regions does not induce memory recall. a-b, A 3-virus cocktail containing c-

Fos-tTA, TRE-Cre, and DIO-ChR2-eYFP was used to achieve randomly labeled (referred to as control) cell populations (**a**). In the home cage and while off a DOX diet, due to leakage of the TRE-Cre viral component enhanced neuronal labeling was achieved. For each brain region (n = 6 mice per group), we first confirmed comparable labeling efficiencies between CFC mice from Fig. 4 and these randomly labeled mice (a 1:3:1 ratio of tTA:TRE:DIO viruses were used for CA1, VLO, and PAG random labeling, a 1:1.5:1 ratio of tTA:TRE:DIO viruses were used for BLA, AHN, and PVT random labeling, a 1:0.7:1 ratio of tTA:TRE:DIO viruses were used for LDTg, RE, and AM random labeling). Subseqently, we performed behavioral activation experiments (**b**). **c**-**k**, eYFP and ChR2-eYFP randomly labeled animals were used for optogenetic reactivation experiments (n = 11 mice per group) targeting CA1 (**c**), BLA (**d**), LDTg (**e**), AHN (**f**), RE (**g**), VLO (**h**), AM (**i**), PAG (**j**), and PVT (**k**). Data are presented as mean ± SEM. Source data are provided as a Source Data file.

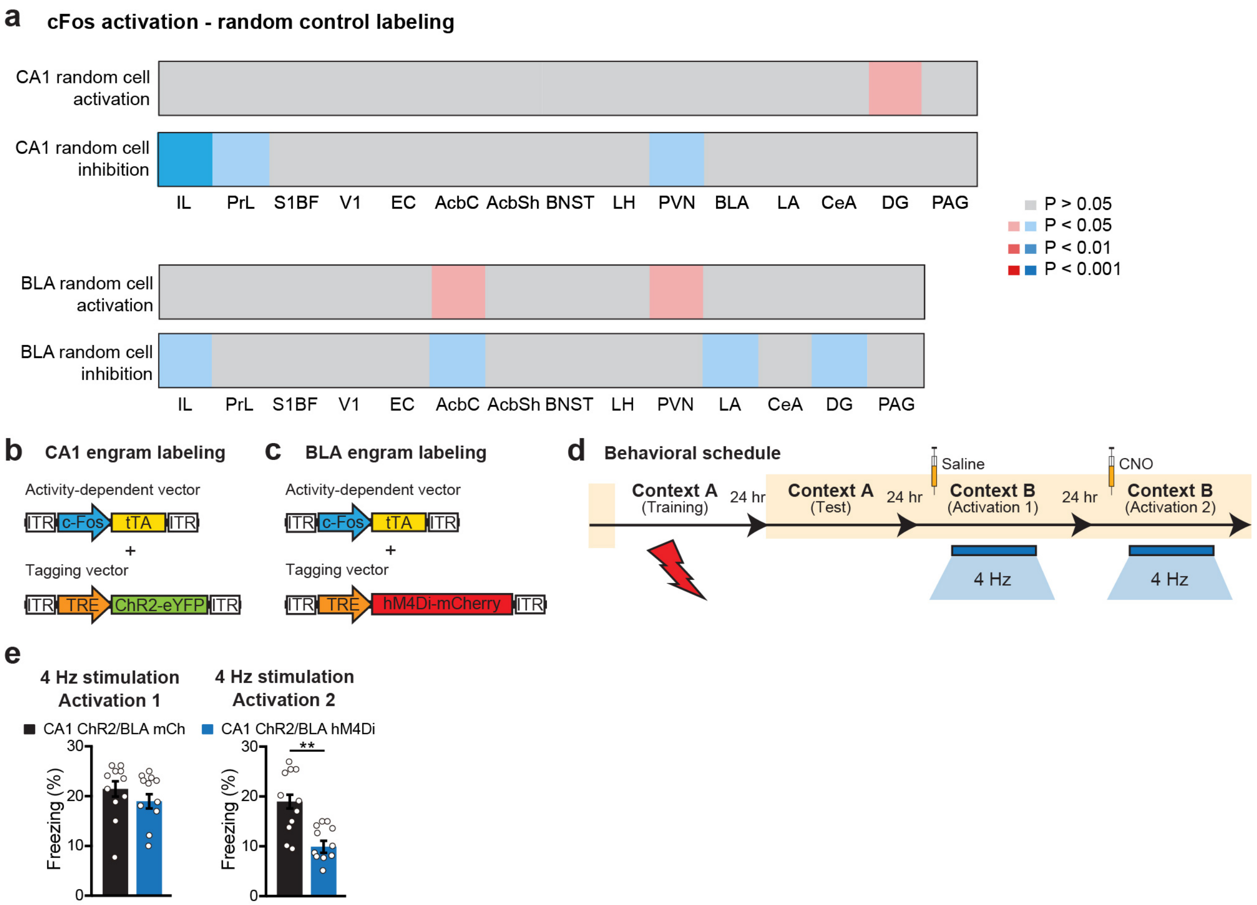

Supplementary Fig. 6. Neural activity following the optogenetic activation of randomly labeled CA1 or BLA ensembles, and CA1 engram cell reactivation with/without simultaneous BLA engram cell inhibition. a, Similar to Fig. 5i and 5s, heat maps representing cFos activation levels across brain regions for randomly labeled ensembles (following strategy in Supplementary Fig. 5a) and their activation (virus cocktail of c-Fos-tTA, TRE-Cre, and DIO-ChR2-eYFP) in a neutral context or inhibition (virus cocktail of c-Fos-tTA, TRE-Cre, and DIO-eArchT-mCherry) during natural memory recall. CA1 (top) and BLA (bottom) (n = 7 mice per group). Red colored regions indicate an increase in the number of cFos$^+$ neurons based on the P value obtained by comparing control vs. manipulation group data, whereas blue colored regions indicate a decrease in the number of cFos$^+$ neurons. **b-e**, Functional connectivity experiment in which CA1 engram cells are reactivated using optogenetics with/without a simultaneous BLA engram cell inhibition using chemogenetics. Virus cocktail of c-Fos-tTA and TRE-ChR2-eYFP was injected into CA1 (**b**), and a virus cocktail of c-Fos-tTA and TRE-hM4Di-mCherry was injected to BLA of the same mice (**c**). Behavior schedule (**d**). In the saline administered Activation 1 test day (i.e., without BLA engram cell inhibition), both CA1 ChR2/BLA mCh and CA1 ChR2/BLA hM4Di groups showed robust optogenetic memory recall, however during the CNO administered Activation 2 test day (i.e., with BLA engram cell inhibition only in the hM4Di group), the BLA hM4Di group showed a significant decrease in optogenetic-induced memory recall as compared to the BLA mCh group (n = 11 mice per group) (**e**). These results indicate that BLA engram cells operate downstream of CA1 engram cells. Statistical

comparisons are performed using two-tailed unpaired t tests; $^{**}P < 0.01$. Data are presented as mean ± SEM. P value: 0.0012 (Activation 2) (**e**). Source data are provided as a Source Data file.

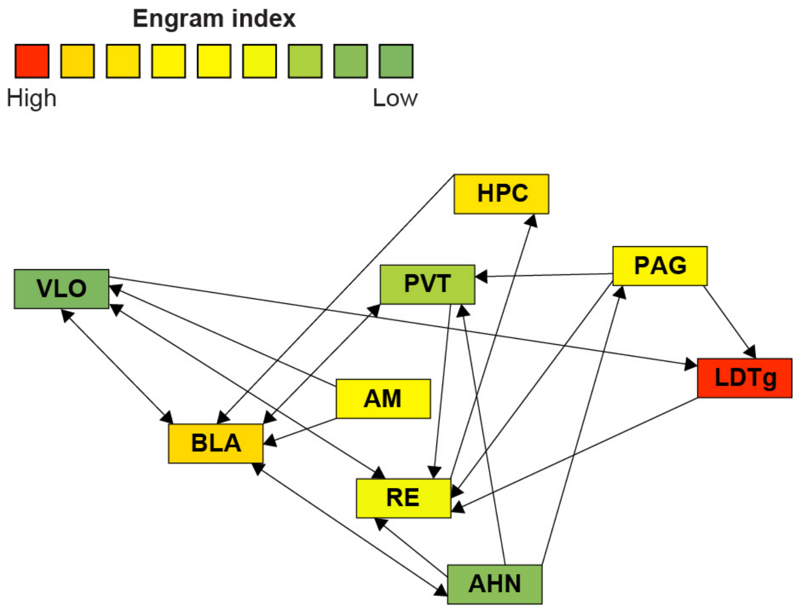

Supplementary Fig. 7. Putative connectivity pattern between engram-holding brain regions identified in this study. For the memory engram regions illustrated in Fig. 7, using the literature we indicated the putative connectivity pattern between these 9 brain regions. We focused on excitatory projections for this figure.

Supplementary Table 1. List of 117 brain regions with statistically significant mean activated neuronal counts in CFC vs. HC and RE vs. HC individually, rank-ordered from high to low engram index values (n = 7 HC mice, n = 10 CFC mice, n = 9 RE mice). A one-way ANOVA followed by Tukey post-hoc test ($P < 0.05$) was used for each region.

	HC		CFC		RE	
**Region name**	Mean	Std. Dev.	Mean	Std. Dev.	Mean	Std. Dev.
Midbrain reticular nucleus	340.00	87.28	689.80	234.97	680.89	340.83
Laterodorsal tegmental nucleus	33.80	9.46	84.45	29.06	82.34	22.93
Presubiculum	140.14	40.58	431.20	175.16	418.44	232.70
Fields of Forel	43.03	27.18	117.43	49.40	120.89	54.98
Cuneiform nucleus	49.86	20.06	90.90	37.08	93.22	30.26
Basolateral amygdalar nucleus, anterior part	87.54	22.21	161.23	53.74	154.22	51.41
Substantia innominata	138.14	32.85	271.20	96.65	285.33	102.86
Nucleus of the lateral olfactory tract, body	35.46	20.91	124.56	66.75	134.56	89.78
Nucleus of the lateral olfactory tract, molecular layer	79.71	27.11	182.40	36.54	170.78	49.97
Medial pretectal area	91.94	12.97	176.98	41.25	165.61	55.82
Tegmental reticular nucleus	150.86	35.27	305.90	80.34	328.11	95.81
Nucleus of the lateral lemniscus	31.00	12.06	75.90	39.07	69.11	27.38
Dentate gyrus	415.43	98.62	927.65	270.65	837.78	416.55
Ectorhinal area	124.57	69.68	354.15	104.11	312.72	208.89
Tuberomammillary nucleus, ventral part	24.47	9.54	49.44	16.83	44.60	18.05
Olivary pretectal nucleus	127.07	31.69	250.55	79.57	225.39	80.91
Superior colliculus, deep white layer	105.68	35.97	357.91	86.97	300.82	49.85
Paraventricular hypothalamic nucleus, descending division	32.02	10.90	108.46	26.35	91.16	1.77
Supraoptic nucleus	96.07	32.70	325.38	79.06	273.47	45.32
Basomedial amygdalar nucleus	423.45	95.67	656.77	123.44	599.65	103.45
Field CA3	305.71	75.32	450.50	90.70	411.00	72.08
Magnocellular reticular nucleus	14.00	9.95	29.40	11.87	33.78	12.66
Basolateral amygdalar nucleus	213.43	63.29	333.40	70.24	298.80	62.09
Claustrum	215.69	65.16	396.63	101.33	341.39	82.06
Lateral habenula	28.26	9.90	84.53	19.90	67.00	21.44
Tuberomammillary nucleus	38.57	16.58	97.06	26.53	78.49	37.86
Field CA1	218.57	90.95	615.52	126.48	487.56	108.92
Frontal pole, layer 6a	213.91	42.08	318.23	62.23	284.20	32.02
Lateral hypothalamic area	99.43	25.17	150.30	33.59	167.11	49.53
Inferior colliculus	2248.57	570.35	4341.70	986.37	3636.11	1350.25
Posterior hypothalamic nucleus	87.14	37.53	170.70	49.08	142.44	41.68
Hippocampo-amygdalar transition area	91.57	16.97	218.62	31.69	174.13	98.79
Ventral premammillary nucleus	27.04	7.57	67.56	23.25	53.31	18.34
Subparafascicular nucleus, magnocellular part	13.52	3.79	33.78	11.63	26.65	9.17
Superior central nucleus raphe	13.52	3.79	33.78	11.63	26.65	9.17
Interanterodorsal nucleus of the thalamus	14.86	4.16	37.12	12.77	29.29	10.08
Trapezoid body	19.31	5.41	48.26	16.61	38.08	13.10
Pontine central gray	34.00	26.10	97.00	37.11	74.44	29.75
Dorsal motor nucleus of the vagus nerve	95.57	38.22	196.70	42.75	160.11	63.29
Midbrain	5955.71	1512.13	10336.70	1831.21	8699.78	2494.62
Olfactory tubercle	30.19	10.18	72.96	24.02	56.96	16.41
Intercalated amygdalar nucleus	12.62	7.09	39.53	14.02	29.41	10.97
Mammillary body	174.57	39.39	297.80	62.49	250.11	57.78
Pedunculopontine nucleus	177.04	41.31	396.33	81.71	311.44	103.09
Endopiriform nucleus, dorsal part	130.00	35.75	356.10	141.72	268.33	95.24
Pallidum	451.29	103.48	1126.30	370.19	859.11	299.85
Periaqueductal gray	225.43	61.59	440.30	95.93	354.00	96.73
Anteromedial thalamic nucleus	66.43	17.95	173.00	47.48	130.00	43.52
Piriform-amygdalar area	152.54	36.70	395.53	75.70	295.36	152.29
Lateral septal nucleus, ventral part	161.71	40.59	558.20	142.34	390.89	183.04
Prosubiculum	39.29	8.49	108.60	33.70	79.22	22.32
Magnocellular nucleus	121.17	45.07	238.55	59.15	188.79	44.25
Temporal association areas	770.43	236.36	2314.70	837.87	1657.22	645.17
Endopiriform nucleus, ventral part	332.86	101.85	853.10	236.09	631.11	244.93
Medial septal nucleus	30.14	8.82	85.30	25.25	61.67	30.30
Basolateral amygdalar nucleus, posterior part	63.44	33.27	129.33	20.11	100.56	33.52
Anteromedial visual area	693.86	344.93	1536.00	454.98	1166.00	178.74
Interanteromedial nucleus of the thalamus	42.66	11.94	87.04	20.98	67.30	15.40
Anterior amygdalar area	29.57	9.62	100.30	28.26	68.44	24.05

Supplementary Table 1 (continued)

Region name	HC		CFC		RE	
	Mean	Std. Dev.	Mean	Std. Dev.	Mean	Std. Dev.
Anteroventral nucleus of thalamus	151.86	51.56	461.00	92.85	603.00	68.86
Nucleus of reuniens	204.71	34.36	368.50	71.29	292.89	79.05
Posterior amygdalar nucleus	282.00	54.73	468.30	86.50	381.44	83.67
Ventral posteromedial nucleus of the thalamus	950.57	333.32	2173.70	396.68	1597.00	630.85
Primary auditory area	1225.29	385.74	3153.70	738.35	2235.89	1010.03
Lateral visual area	732.29	227.54	1824.00	499.35	1303.89	382.22
Posterior pretectal nucleus	90.14	23.71	270.00	99.99	183.94	62.88
Taenia tecta, ventral part	64.86	18.67	179.60	54.84	123.67	36.18
Subparafascicular nucleus	5.35	1.30	13.01	2.80	9.28	2.66
Parataenial nucleus	53.54	13.02	130.12	37.99	92.77	26.56
Central amygdalar nucleus, medial part	61.05	12.18	189.73	55.96	124.96	58.46
Ventral group of the dorsal thalamus	1539.57	513.83	3539.40	702.24	2525.11	841.87
Globus pallidus, external segment	90.00	28.08	230.00	56.16	158.00	63.54
Ventromedial hypothalamic nucleus	59.43	19.87	168.90	33.41	111.89	44.82
Paraventricular nucleus of the thalamus	209.57	59.06	586.10	163.86	388.33	135.22
Cerebral nuclei	2538.29	576.12	6532.70	1250.55	4427.00	1230.62
Postrhinal area	342.57	103.24	1003.90	231.88	644.22	213.99
Epithalamus	42.43	18.49	162.40	53.22	97.00	36.16
Medial amygdalar nucleus	347.71	151.80	1183.60	318.43	724.44	254.71
Medial habenula	77.59	18.61	149.63	34.18	109.78	16.32
Striatum	2087.00	474.02	5406.40	720.52	3567.89	788.96
Brain stem	17665.71	4577.44	33430.50	3953.92	24629.56	5882.23
Cortical subplate	1380.14	395.74	2680.90	457.48	1951.56	453.68
Tuberal nucleus	11.13	4.17	37.33	7.13	22.63	11.34
Anterior hypothalamic nucleus	126.07	32.70	325.38	79.06	213.47	65.32
Diagonal band nucleus	25.21	6.54	65.08	15.81	42.69	13.06
Paraventricular hypothalamic nucleus	84.05	21.80	216.92	52.71	142.32	43.54
Orbital area, ventrolateral part	631.24	222.89	1882.33	466.19	1170.44	354.63
Cortical amygdalar area, posterior part	372.86	162.56	1328.70	377.82	781.67	309.13
Piriform area	3402.00	1099.77	7949.70	1452.32	5331.78	1317.94
Infralimbic area	230.51	82.27	585.76	63.22	380.90	80.90
Hypothalamic medial zone	202.86	119.44	783.10	192.13	448.33	103.30
Nucleus accumbens	239.57	85.84	589.90	91.24	383.78	95.15
Basolateral amygdalar nucleus, ventral part	27.78	14.53	84.56	18.19	50.89	15.96
Striatum-like amygdalar nuclei	501.86	181.34	1638.50	330.92	963.78	363.08
Entorhinal area, lateral part	892.71	320.70	2575.10	417.25	1575.00	325.99
Basomedial amygdalar nucleus, posterior part	258.00	103.76	589.30	109.70	391.89	85.87
Posterior parietal association areas	1611.86	540.20	4855.10	1096.77	2914.56	981.60
Fundus of striatum	146.61	32.43	436.08	75.74	262.36	89.12
Caudoputamen	586.43	129.73	1744.30	382.97	1049.44	436.48
Posterior intralaminar thalamic nucleus	92.33	33.05	259.20	70.86	157.24	30.97
Dorsal peduncular area	60.03	15.48	149.71	27.72	94.43	25.32
Postsubiculum	614.86	198.53	1683.10	385.75	1022.89	249.44
Prelimbic area	224.76	74.04	522.64	90.98	337.67	92.48
Orbital area, medial part	372.71	122.62	1153.90	192.24	662.33	263.09
Orbital area, lateral part	1449.00	502.62	4132.40	818.06	2441.33	780.01
Retrosplenial area, dorsal part	3168.86	218.33	5088.40	570.05	3852.22	612.21
Agranular insular area, ventral part	393.00	123.82	1050.20	203.32	618.44	171.01
Retrohippocampal region	2653.43	792.37	8268.10	1849.36	4527.00	1165.34
Dorsal cochlear nucleus	100.57	20.08	262.50	114.30	152.56	99.43
Thalamus	4740.57	1489.74	12825.80	2080.01	7292.11	2148.97
Anterior cingulate area, dorsal part	1462.00	495.17	4204.90	729.03	2308.00	666.24
Anterior cingulate area, ventral part	748.14	249.05	2850.60	695.75	1395.22	405.20
Perirhinal area	242.55	70.37	616.71	83.59	354.58	96.73
Entorhinal area, medial part	539.86	179.79	2145.20	335.69	986.22	273.26
Thalamus, polymodal association cortex related	2371.71	707.12	7786.20	1411.46	3801.00	868.08
Midline group of the dorsal thalamus	317.71	80.01	965.30	131.12	484.22	159.89
Mediodorsal nucleus of thalamus	267.86	113.44	1210.00	182.79	506.00	225.55

Supplementary Table 2. List of 130 brain regions that did not have statistically significant mean activated neuronal counts in CFC vs. HC or RE vs. HC individually, rank-ordered alphabetically (n = 7 HC mice, n = 10 CFC mice, n = 9 RE mice). A one-way ANOVA followed by Tukey post-hoc test (P > 0.05) was used for each region.

	HC		CFC		RE	
**Region name**	Mean	Std. Dev.	Mean	Std. Dev.	Mean	Std. Dev.
Accessory olfactory bulb	135.29	45.23	179.30	49.28	135.44	99.62
Agranular insular area, dorsal part	1017.29	369.30	2059.80	641.29	1056.44	639.13
Agranular insular area, posterior part	391.29	147.17	877.20	446.45	428.78	240.84
Ansiform lobule	2328.71	360.92	3901.50	678.77	2476.00	1123.43
Anterior group of the dorsal thalamus	460.29	149.84	1634.20	495.91	790.22	430.43
Anterior olfactory nucleus	1724.71	612.55	3250.40	902.08	2263.67	1242.26
Anterior pretectal nucleus	263.71	77.79	486.80	134.86	399.00	188.22
Anterodorsal thalamic nucleus	57.14	14.34	79.80	26.27	63.11	47.33
Anterolateral visual area	57.86	13.02	121.80	53.13	87.56	57.00
Anteromedial nucleus, dorsal part	175.86	49.97	384.00	154.77	318.89	140.94
Arcuate hypothalamic nucleus	84.14	14.78	287.70	135.20	86.33	66.21
Area prostriata	62.14	10.40	92.80	31.94	73.22	50.20
Basomedial amygdalar nucleus, anterior part	195.86	55.29	417.30	165.77	243.89	128.64
Bed nuclei of the stria terminalis	282.71	60.38	371.20	92.20	274.11	138.88
Central amygdalar nucleus	91.57	18.26	284.60	113.94	187.44	87.68
Central amygdalar nucleus, capsular part	30.52	6.09	94.87	37.98	62.48	29.23
Central lateral nucleus of the thalamus	137.29	31.12	210.90	64.02	194.78	83.61
Central lobule	1377.29	195.29	2862.90	489.61	2526.78	1614.09
Central medial nucleus of the thalamus	169.43	48.98	253.20	67.75	262.33	123.85
Cerebellar cortex	13479.71	2183.98	23274.20	3609.25	16732.44	7898.55
Cerebellar nuclei	126.86	31.92	157.50	53.22	138.00	85.17
Cerebellum	13727.14	2215.70	23580.30	3658.46	17015.44	8012.68
Cerebral cortex	80204.57	25496.93	190839.40	29196.48	119653.22	54836.15
Cerebrum	82742.86	26036.22	197372.10	30750.11	124080.22	56453.86
Cortical amygdalar area, anterior part	468.57	147.24	822.40	426.92	428.67	240.86
Cortical plate	78824.43	25104.17	188158.50	28595.21	118001.67	54183.53
Crus 1	1379.71	203.94	2306.60	406.88	1500.22	652.19
Crus 2	949.00	170.64	1594.90	517.01	975.78	526.13
Culmen	2339.14	420.78	3182.30	622.33	2842.00	1520.17
Cuneate nucleus	42.29	15.41	44.30	22.06	52.11	31.11
Declive	1152.29	208.32	1646.70	373.77	1260.22	682.38
Dorsal column nuclei	28.57	5.91	58.30	20.92	30.44	17.49
Dorsal part of the lateral geniculate complex	393.00	172.62	729.90	335.10	383.56	242.92
Dorsomedial nucleus of the hypothalamus	169.14	71.89	182.70	65.02	173.78	72.40
External cuneate nucleus	66.14	16.14	137.20	85.82	119.00	70.80
Flocculus	269.43	76.05	453.80	234.04	194.56	169.67
Frontal pole, cerebral cortex	482.43	143.78	769.70	228.31	633.67	321.94
Frontal pole, layer 2/3	387.93	93.03	748.15	170.90	548.89	211.60
Frontal pole, layer 5	103.71	31.07	260.40	112.82	180.33	87.26
Geniculate group, dorsal thalamus	570.14	217.40	1022.20	343.00	612.44	355.02
Gigantocellular reticular nucleus	170.14	57.49	198.00	94.24	272.56	167.21
Gustatory areas	200.29	30.12	309.60	108.58	271.00	92.96
Hindbrain	5646.14	1365.76	7676.00	1602.83	7065.22	2847.67
Intermediate reticular nucleus	281.71	87.74	284.30	106.39	406.00	226.74
Intermediodorsal nucleus of the thalamus	33.48	14.18	121.25	35.35	63.25	40.69
Intralaminar nuclei of the dorsal thalamus	423.00	139.48	1193.00	294.89	696.33	374.58
Isocortex	59888.86	19698.51	144370.00	19137.46	91426.00	42125.99
Lateral amygdalar nucleus	175.43	55.72	329.50	170.26	264.67	176.97
Lateral dorsal nucleus of thalamus	117.14	42.79	299.90	123.25	112.89	46.62
Lateral group of the dorsal thalamus	489.57	170.26	1660.00	489.43	590.78	368.16
Lateral posterior nucleus of the thalamus	167.43	54.15	704.60	340.50	211.89	137.09
Lateral reticular nucleus	82.29	19.80	174.30	56.83	146.56	93.36
Main olfactory bulb	7072.57	2466.37	15741.40	6480.94	8760.78	6740.95
Medial geniculate complex, medial part	87.29	25.26	138.30	66.24	96.44	36.03
Medial geniculate complex, ventral part	125.00	32.27	160.50	64.94	159.78	66.26
Medial group of the dorsal thalamus	399.29	150.88	1726.50	390.81	760.67	491.16
Medial mammillary nucleus	122.71	30.89	178.80	90.21	141.44	172.11
Medial preoptic nucleus	46.43	11.75	49.60	24.18	41.44	20.96
Medial vestibular nucleus	203.86	45.39	273.10	103.93	296.78	188.02
Medulla	3992.00	978.92	5296.20	1411.37	5016.89	2154.03
Medulla, motor related	2042.43	569.39	2288.90	622.53	2527.44	1315.93
Medulla, sensory related	1436.29	330.39	2387.80	830.20	1880.44	824.65
Medullary reticular nucleus	411.71	136.99	296.40	248.95	472.56	420.03
Midbrain raphe nuclei	103.57	31.17	93.10	92.43	114.44	64.39

Supplementary Table 2 (continued)

	HC		CFC		RE	
Region name	Mean	Std. Dev.	Mean	Std. Dev.	Mean	Std. Dev.
Midbrain, behavioral state related	111.71	20.79	391.80	189.99	113.78	74.96
Midbrain, motor related	2181.86	564.02	3618.80	704.18	3005.56	1130.01
Midbrain, sensory related	2742.57	689.34	5167.80	1005.32	4299.44	1938.67
Nodulus	482.86	93.34	686.30	268.44	749.33	534.95
Nucleus of the solitary tract	118.86	38.03	179.80	88.28	133.67	64.55
Olfactory areas	15156.57	4665.06	20166.33	11209.88	32978.80	10887.72
Parabrachial nucleus	138.86	31.81	203.20	42.62	145.89	65.66
Paracentral nucleus	57.86	9.02	116.50	37.68	86.56	28.05
Parafascicular nucleus	66.71	13.10	94.40	35.60	74.22	30.14
Paraflocculus	468.29	166.70	1106.70	596.55	362.11	428.90
Paragigantocellular reticular nucleus	151.29	43.54	131.50	90.40	153.89	84.58
Parasubiculum	109.71	31.16	403.40	161.16	190.67	131.76
Parvicellular reticular nucleus	267.57	90.82	303.00	111.22	341.44	167.01
Periventricular region	149.29	35.45	309.70	181.89	134.00	48.23
Pons, behavioral state related	122.86	43.45	228.90	143.06	130.56	56.56
Pons, motor related	803.29	213.85	938.70	514.02	953.56	445.37
Pons, sensory related	430.43	92.40	774.60	157.23	594.00	212.78
Pontine reticular nucleus	84.14	33.86	155.00	108.98	82.56	41.19
Posterior auditory area	265.43	116.48	302.60	400.81	287.56	165.13
Posterior complex of the thalamus	126.57	38.33	198.30	60.90	181.67	111.60
Posterior limiting nucleus of the thalamus	289.57	115.83	211.40	212.92	326.89	272.26
Posterior triangular thalamic nucleus	326.14	64.17	474.20	191.29	445.22	258.83
Posterolateral visual area	346.29	85.25	555.60	198.65	402.44	194.62
Postpiriform transition area	100.86	26.16	260.30	87.25	122.78	100.25
Primary motor area	1521.90	562.12	3500.23	906.06	2301.59	1381.18
Primary somatosensory area, barrel field	3638.00	1205.63	10052.40	2360.57	6432.00	2788.38
Primary somatosensory area, lower limb	1014.14	341.09	3263.90	972.14	1989.22	1194.65
Primary somatosensory area, mouth	4365.57	1539.87	8332.20	2274.49	5441.56	2921.71
Primary somatosensory area, nose	1693.57	572.27	4481.30	1028.62	3036.56	1431.56
Primary somatosensory area, trunk	527.71	188.16	1922.60	465.54	1116.56	688.48
Primary somatosensory area, upper limb	2127.86	859.83	5627.00	1513.22	3154.56	1678.08
Primary visual area	5407.43	1690.29	13014.40	3219.58	8056.44	4144.07
Pyramus	639.00	126.01	1166.30	579.38	820.22	464.83
Reticular nucleus of the thalamus	70.00	20.58	191.60	91.17	119.44	60.41
Retrosplenial area, ventral part	3018.29	1005.30	5264.10	1188.83	3803.22	2140.04
Rhomboid nucleus	132.71	41.00	250.40	73.01	153.11	71.35
Rostrolateral visual area	218.43	99.83	444.90	161.68	212.33	132.47
Secondary motor area	5009.57	1871.90	11532.60	2319.98	7701.89	4086.24
Somatosensory areas	18124.71	6210.30	46550.30	8888.46	29309.67	13578.23
Spinal nucleus of the trigeminal, caudal part	466.14	138.68	846.10	575.52	502.11	249.86
Spinal nucleus of the trigeminal, interpolar part	260.71	64.95	372.80	142.14	461.78	352.03
Spinal vestibular nucleus	193.43	46.31	265.50	81.13	163.67	76.72
Subiculum	149.43	50.80	441.60	383.96	421.78	351.36
Submedial nucleus of the thalamus	210.29	49.58	262.40	65.06	207.67	125.67
Substantia nigra, reticular part	88.29	33.58	156.70	91.11	134.22	72.72
Superior colliculus, deep gray layer	465.86	107.03	646.40	141.82	515.11	266.84
Superior colliculus, motor related	876.14	230.17	1381.80	386.52	1069.78	432.16
Superior colliculus, optic layer	72.86	20.27	214.00	91.38	136.11	81.10
Superior colliculus, superficial gray layer	284.57	87.39	457.60	172.07	386.89	199.16
Superior olivary complex	154.86	40.05	176.70	73.69	178.44	78.41
Supplemental somatosensory area	4111.43	1354.92	10794.80	3203.03	6906.44	3246.40
Supramammillary nucleus	111.14	32.75	104.90	44.61	104.89	49.56
Taenia tecta, dorsal part	149.79	49.04	259.05	115.57	171.39	98.33
Thalamus, sensory-motor cortex related	2187.86	743.13	4662.10	789.65	3236.89	1492.21
Uvula	1028.71	168.20	1454.90	604.76	1294.22	745.62
Ventral anterolateral complex of the thalamus	155.71	35.20	304.80	155.02	181.56	112.74
Ventral auditory area	813.00	246.62	1994.30	582.91	1505.22	706.43
Ventral cochlear nucleus	225.57	51.95	211.70	92.96	292.67	182.11
Ventral medial nucleus of the thalamus	105.57	41.43	247.30	78.06	183.67	176.46
Ventral part of the lateral geniculate complex	147.43	42.24	218.20	61.69	222.78	103.90
Ventral posterior complex of the thalamus	1173.57	399.54	2650.40	495.87	1929.00	850.90
Ventral posterolateral nucleus of the thalamus	154.43	27.80	210.30	78.06	191.67	109.37
Ventral posteromedial nucleus of the thalamus, parvicellular part	316.86	111.11	724.57	132.23	532.33	243.62
Vermal regions	7555.14	1196.08	11986.00	2524.64	10080.00	5612.87
Visceral area	62.86	17.00	86.20	38.24	81.11	39.38
Zona incerta	268.71	101.57	353.40	100.07	310.44	207.33

Supplementary Table 3. List of 159 brain regions that did not have statistically significant engram reactivation relative to chance level, rank-ordered from high to low engram reactivation values (i.e., percentage of learning-activated neurons that showed recall-induced cFos). A two-tailed one-sample *t* test (P > 0.05) was used for each region. Behaviorally-tested regions are labeled with an asterisk.

a

Engram reactivation	
12.44	Lateral reticular nucleus
12.24	Anterolateral visual area
12.24	Orbital area, lateral part
12.17	Basomedial amygdalar nucleus, anterior part
12.14	Rostrolateral visual area
12.02	Superior colliculus, superficial gray layer
12.00	Tegmental reticular nucleus
11.84	Taenia tecta, dorsal part
11.81	Postrhinal area
11.76	Subiculum
11.75	Anterior olfactory nucleus
11.74	Entorhinal area, medial part
11.73	External cuneate nucleus
11.44	Frontal pole, layer 5
11.37	Posterior parietal association areas
11.24	Parafascicular nucleus
11.07	Piriform area
10.97	Central medial nucleus of the thalamus
10.93	Cortical amygdalar area, anterior part
10.83	Periventricular region
10.72	Temporal association areas
* 10.64	Primary visual area
10.47	Central amygdalar nucleus, capsular part
10.35	Lateral amygdalar nucleus
9.91	Pons, motor related
9.63	Parabrachial nucleus
9.61	Intermediodorsal nucleus of the thalamus
9.59	Substantia nigra, reticular part
9.56	Retrosplenial area, dorsal part
9.47	Ventral posteromedial nucleus of the thalamus, parvicellular part
9.42	Postpiriform transition area
9.35	Taenia tecta, ventral part
9.35	Pons, behavioral state related
9.35	Pallidum
9.28	Midbrain, behavioral state related
9.23	Superior olivary complex
9.10	Posterolateral visual area
9.06	Ventral part of the lateral geniculate complex
9.03	Retrohippocampal region
8.73	Paragigantocellular reticular nucleus
8.72	Agranular insular area, posterior part
* 8.45	Mediodorsal nucleus of thalamus
8.29	Ventral auditory area
8.23	Central amygdalar nucleus, medial part
8.18	Superior colliculus, optic layer
8.15	Field CA3
8.13	Cerebrum
8.06	Supramammillary nucleus
8.03	Posterior triangular thalamic nucleus
7.99	Cerebral cortex
7.98	Frontal pole, cerebral cortex
7.97	Secondary motor area
7.89	Rhomboid nucleus
7.89	Cortical plate
7.89	Dorsomedial nucleus of the hypothalamus
7.87	Arcuate hypothalamic nucleus
7.87	Isocortex
7.83	Olfactory areas
7.79	Globus pallidus, external segment
7.64	Nucleus of the solitary tract
7.60	Posterior auditory area
7.51	Medial geniculate complex, ventral part
7.37	Midbrain raphe nuclei
7.19	Medial preoptic nucleus
7.15	Agranular insular area, dorsal part
7.14	Dorsal column nuclei
7.11	Anteromedial nucleus, dorsal part
6.97	Zona incerta
6.93	Superior colliculus, deep gray layer
6.84	Medial geniculate complex, medial part
6.79	Medial group of the dorsal thalamus
6.78	Intralaminar nuclei of the dorsal thalamus
6.78	Midbrain, motor related
6.77	Medial vestibular nucleus
6.73	Posterior limiting nucleus of the thalamus
6.62	Parasubiculum
6.60	Presubiculum
6.60	Reticular nucleus of the thalamus
6.58	Midbrain
6.53	Gigantocellular reticular nucleus
6.45	Central amygdalar nucleus
6.41	Thalamus, polymodal association cortex related

(continued on top of panel b)

b

Engram reactivation	
6.44	Accessory olfactory bulb
6.44	Superior colliculus, motor related
6.41	Pontine reticular nucleus
6.32	Intermediate reticular nucleus
6.32	Ventral cochlear nucleus
6.28	Spinal vestibular nucleus
6.24	Gustatory areas
6.18	Frontal pole, layer 2/3
6.13	Mammillary body
6.07	Cuneate nucleus
6.05	Brain stem
5.90	Midbrain, sensory related
5.86	Primary auditory area
5.73	Lateral dorsal nucleus of thalamus
5.72	Hindbrain
5.72	Cerebellar nuclei
5.54	Bed nuclei of the stria terminalis
* 5.39	Primary somatosensory area, barrel field
5.35	Medulla, motor related
5.34	Anterodorsal nucleus
5.26	Inferior colliculus
5.25	Paracentral nucleus
5.18	Thalamus
5.17	Midbrain reticular nucleus
5.10	Crus 1
5.01	Visceral area
5.00	Primary somatosensory area, lower limb
5.00	Primary somatosensory area, trunk
4.97	Paraflocculus
4.86	Parvicellular reticular nucleus
4.65	Ansiform lobule
4.56	Dorsal cochlear nucleus
4.47	Central lateral nucleus of the thalamus
4.45	Anterior group of the dorsal thalamus
4.42	Primary motor area
4.41	Medulla
4.40	Supplemental somatosensory area
4.37	Medial mammillary nucleus
4.34	Culmen
4.32	Pons, sensory related
4.30	Retrosplenial area, ventral part
4.30	Ventral medial nucleus of the thalamus
4.20	Main olfactory bulb
4.18	Crus 2
4.08	Primary somatosensory area, upper limb
3.94	Medullary reticular nucleus
3.89	Flocculus
3.89	Somatosensory areas
3.88	Central lobule
3.78	Posterior complex of the thalamus
3.77	Cerebellar cortex
3.67	Vermal regions
3.61	Declive
3.59	Lateral posterior nucleus of the thalamus
3.54	Spinal nucleus of the trigeminal, interpolar part
3.50	Nodulus
3.44	Cerebellum
3.22	Submedial nucleus of the thalamus
3.15	Uvula
3.13	Pyramus
3.08	Anterior pretectal nucleus
2.98	Medulla, sensory related
2.76	Geniculate group, dorsal thalamus
2.75	Ventral anterolateral complex of the thalamus
2.72	Ventral posterolateral nucleus of the thalamus
2.66	Lateral group of the dorsal thalamus
2.56	Area prostriata
2.10	Anteroventral nucleus of thalamus
2.02	Dorsal part of the lateral geniculate complex
1.98	Postsubiculum
1.85	Thalamus, sensory-motor cortex related
1.85	Primary somatosensory area, nose
1.69	Primary somatosensory area, mouth
1.34	Ventral group of the dorsal thalamus
1.33	Spinal nucleus of the trigeminal, caudal part
1.23	Ventral posterior complex of the thalamus
1.11	Ventral posteromedial nucleus of the thalamus
