# Supplementary material for: Brain-wide mapping reveals that engrams for a single memory are distributed across multiple brain regions
Source: Nat Commun. 2022 Apr 4;13:1799. doi: 10.1038/s41467-022-29384-4 (PMC8980018; doi:10.1038/s41467-022-29384-4)
Supplement: Supplementary file 5 — Reporting Summary [file 41467_2022_29384_MOESM5_ESM.pdf]

nature portfolio

Corresponding author(s): Susumu Tonegawa

Last updated by author(s): Mar 1, 2022

Reporting Summary

Nature Portfolio wishes to improve the reproducibility of the work that we publish. This form provides structure for consistency and transparency in reporting. For further information on Nature Portfolio policies, see our Editorial Policies and the Editorial Policy Checklist.

Statistics

For all statistical analyses, confirm that the following items are present in the figure legend, table legend, main text, or Methods section.

n/a	Confirmed	
☐	☒	The exact sample size (*n*) for each experimental group/condition, given as a discrete number and unit of measurement
☐	☒	A statement on whether measurements were taken from distinct samples or whether the same sample was measured repeatedly
☐	☒	The statistical test(s) used AND whether they are one- or two-sided<br>*Only common tests should be described solely by name; describe more complex techniques in the Methods section.*
☒	☐	A description of all covariates tested
☐	☒	A description of any assumptions or corrections, such as tests of normality and adjustment for multiple comparisons
☐	☒	A full description of the statistical parameters including central tendency (e.g. means) or other basic estimates (e.g. regression coefficient) AND variation (e.g. standard deviation) or associated estimates of uncertainty (e.g. confidence intervals)
☐	☒	For null hypothesis testing, the test statistic (e.g. *F*, *t*, *r*) with confidence intervals, effect sizes, degrees of freedom and *P* value noted<br>*Give P values as exact values whenever suitable.*
☒	☐	For Bayesian analysis, information on the choice of priors and Markov chain Monte Carlo settings
☒	☐	For hierarchical and complex designs, identification of the appropriate level for tests and full reporting of outcomes
☐	☒	Estimates of effect sizes (e.g. Cohen's *d*, Pearson's *r*), indicating how they were calculated

Our web collection on statistics for biologists contains articles on many of the points above.

Software and code

Policy information about availability of computer code

Data collection	Light-sheet microscopy and brain-wide quantitative activity mapping used TeraStitcher, Imaris (version 9.5), and custom-generated MATLAB scripts. Fear conditioning behavior used the FreezeFrame (version 4) software. Manual cell counting used ImageJ (version 1.53) software.
Data analysis	Data analysis used Prism (version 6) and Microsoft Excel programs. Custom code generated for this study is available on GitHub: https://github.com/chunglabmit/bmtrap_2021.

For manuscripts utilizing custom algorithms or software that are central to the research but not yet described in published literature, software must be made available to editors and reviewers. We strongly encourage code deposition in a community repository (e.g. GitHub). See the Nature Portfolio guidelines for submitting code & software for further information.

Data

Policy information about availability of data

All manuscripts must include a data availability statement. This statement should provide the following information, where applicable:

- Accession codes, unique identifiers, or web links for publicly available datasets
- A description of any restrictions on data availability
- For clinical datasets or third party data, please ensure that the statement adheres to our policy

Supplementary Information/Source Data are provided with this paper. Whole brain imaging data is available from the laboratory of Prof. Kwanghun Chung upon reasonable request.

Field-specific reporting

Please select the one below that is the best fit for your research. If you are not sure, read the appropriate sections before making your selection.

☒ Life sciences ☐ Behavioural & social sciences ☐ Ecological, evolutionary & environmental sciences

For a reference copy of the document with all sections, see nature.com/documents/nr-reporting-summary-flat.pdf

Life sciences study design

All studies must disclose on these points even when the disclosure is negative.

Sample size	For brain-wide experimental analyses, we used an n = 7-10 mice per group, which is consistent with the sample size used in the literature (e.g., DeNardo et al., Nat Neurosci 2019). Similarly, we used at least n = 10 mice per group for all behavioral manipulation experiments (i.e., optogenetic and chemogenetic studies), consistent with the literature (e.g., Hong et al., Cell 2014).
Data exclusions	Exclusion criteria were pre-established. Following behavioral experiments, only animals with efficient viral labeling in the target region were used for subsequent behavioral data quantification.
Replication	All experiments were performed in at least two separate batches to confirm that the observed phenotypes in any one batch can be replicated. These attempts were successful.
Randomization	Transgenic mice for brain-wide labeling studies as well as surgery mice for manipulation studies were randomly assigned to the different behavioral groups for each experimental type.
Blinding	Investigators were blind to the experimental group information during data collection and analyses.

Reporting for specific materials, systems and methods

We require information from authors about some types of materials, experimental systems and methods used in many studies. Here, indicate whether each material, system or method listed is relevant to your study. If you are not sure if a list item applies to your research, read the appropriate section before selecting a response.

Materials & experimental systems

n/a	Involved in the study	
☐	☒	Antibodies
☒	☐	Eukaryotic cell lines
☒	☐	Palaeontology and archaeology
☐	☒	Animals and other organisms
☒	☐	Human research participants
☒	☐	Clinical data
☒	☐	Dual use research of concern

Methods

n/a	Involved in the study	
☒	☐	ChIP-seq
☒	☐	Flow cytometry
☒	☐	MRI-based neuroimaging

Antibodies

Antibodies used	cFos (ab214672, clone EPR20769, Abcam), dye-conjugated Fc-specific Fab fragments for secondary antibody staining (2:1 molar ratio to the primary antibody, 705-547-003, Jackson ImmunoResearch); chicken anti-GFP (A10262, Life Technologies), anti-chicken Alexa-488 (A11039, Invitrogen); rabbit anti-RFP (600-401-379, Rockland Inc.), anti-rabbit Alexa-555 (A21428, Invitrogen); cFos in sections was stained with rabbit anti-cFos (sc-166940, clone E-8, Santa Cruz); for counterstaining in Fig. 5, sections were incubated with Neuro Trace Fluorescent Nissl Stain (N21483, Molecular Probes); for chance level in Fig. 3, mouse anti-NeuN (MAB377, clone A60, Millipore) was used.
Validation	All these antibodies have been validated by the manufacturer and other researchers using cell lines, western blots, and mouse brain tissue.

Animals and other organisms

Policy information about studies involving animals; ARRIVE guidelines recommended for reporting animal research

Laboratory animals	The C57BL/6J wild type male mice were obtained from Jackson Laboratory. For brain-wide neural activity labeling based on the c-fos promoter, we used the previously described c-fos-CreERT2 mouse line. For brain-wide labeling experiments, FosTRAP mice were crossed with the Cre-dependent tdTomato reporter mouse line Ai14, which were obtained from Jackson Laboratory (Stock No. 007908). All mouse lines were maintained as hemizygotes. For behavioral and brain-wide labeling experiments, all mice were male and 3-6 months old. All animals were housed in a 7 am-7pm light on-off cycle facility with a temperature of 18-23 degrees C and

	humidity maintained between 40-60%.
Wild animals	No wild animals were used in the study.
Field-collected samples	No field collected samples were used in the study.
Ethics oversight	All experiments were conducted in accordance with U.S. National Institutes of Health (NIH) guidelines and the Massachusetts Institute of Technology Department of Comparative Medicine and Committee of Animal Care.

Note that full information on the approval of the study protocol must also be provided in the manuscript.
